# SETDB1 Links the Meiotic DNA Damage Response to Sex Chromosome Silencing in Mice

**DOI:** 10.1016/j.devcel.2018.10.004

**Published:** 2018-12-03

**Authors:** Takayuki Hirota, Paul Blakeley, Mahesh N. Sangrithi, Shantha K. Mahadevaiah, Vesela Encheva, Ambrosius P. Snijders, Elias ElInati, Obah A. Ojarikre, Dirk G. de Rooij, Kathy K. Niakan, James M.A. Turner

**Affiliations:** 1 Sex Chromosome Biology Laboratory, The Francis Crick Institute, London NW1 1AT, UK; 2 Human Embryo and Stem Cell Laboratory, The Francis Crick Institute, London NW1 1AT, UK; 3 KK Women’s and Children’s Hospital, Department of Reproductive Medicine, Singapore 229899, Singapore; 4 Duke-NUS Graduate Medical School, Singapore 119077, Singapore; 5 Mass Spectrometry Science Technology Platform, The Francis Crick Institute, London NW1 1AT, UK; 6 Reproductive Biology Group, Division of Developmental Biology, Department of Biology, Faculty of Science, Utrecht University, Utrecht 3584 CH, the Netherlands; 7 Center for Reproductive Medicine, Academic Medical Center, University of Amsterdam, Amsterdam 1105 AZ, the Netherlands

**Keywords:** meiotic silencing, MSCI, sex chromosomes, DNA damage response, mouse, H3K9me3

## Abstract

Meiotic synapsis and recombination ensure correct homologous segregation and genetic diversity. Asynapsed homologs are transcriptionally inactivated by meiotic silencing, which serves a surveillance function and in males drives meiotic sex chromosome inactivation. Silencing depends on the DNA damage response (DDR) network, but how DDR proteins engage repressive chromatin marks is unknown. We identify the histone H3-lysine-9 methyltransferase SETDB1 as the bridge linking the DDR to silencing in male mice. At the onset of silencing, X chromosome H3K9 trimethylation (H3K9me3) enrichment is downstream of DDR factors. Without *Setdb1*, the X chromosome accrues DDR proteins but not H3K9me3. Consequently, sex chromosome remodeling and silencing fail, causing germ cell apoptosis. Our data implicate TRIM28 in linking the DDR to SETDB1 and uncover additional factors with putative meiotic XY-silencing functions. Furthermore, we show that SETDB1 imposes timely expression of meiotic and post-meiotic genes. *Setdb1* thus unites the DDR network, asynapsis, and meiotic chromosome silencing.

## Highlights

• The histone methyltransferase SETDB1 is essential for male mouse meiosis
• The meiotic DDR network recruits SETDB1 to the XY pair, where it induces H3K9me3
• SETDB1 deletion perturbs meiotic sex chromosome remodeling and silencing
• SETDB1 ensures timely expression of meiotic and post-meiotic genes

## Graphical Abstract

**Figure:**
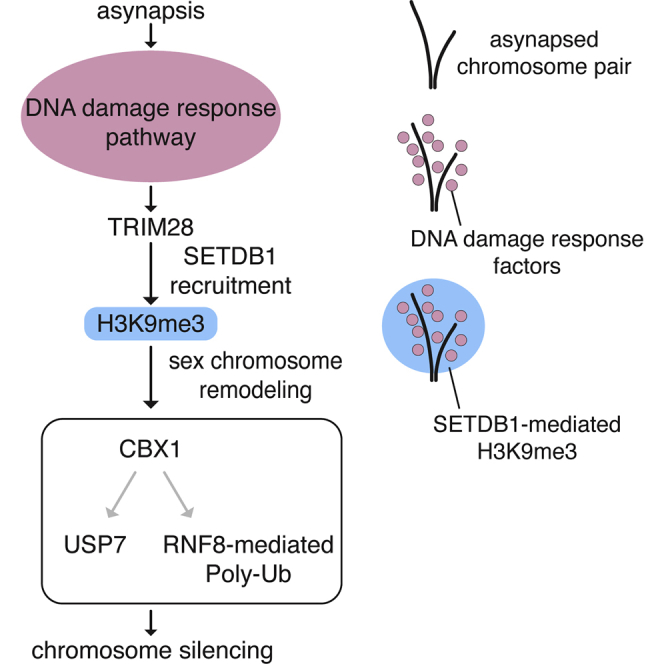

## Teaser

During male meiosis in mammals, the asynapsed regions of the X and Y chromosomes retain DNA double-strand breaks (DSBs), which triggers silencing of the sex chromosomes, a process essential for fertility. Hirota et al. show that meiotic DSB factors recruit the H3K9-methyltransferase SETDB1, inducing XY-chromatin remodeling and silencing.

## Introduction

Defective synapsis or recombination can cause mutation and aneuploidy in offspring. To prevent these outcomes, surveillance mechanisms operate during prophase I to eliminate germ cells in which either process is defective. Current data support the existence in mice of two such surveillance mechanisms. The first is triggered by persistent DNA damage and transduces germ cell elimination via the CHK2/p53/p63 checkpoint pathway (Bolcun-Filas et al., 2014, Di Giacomo et al., 2005, Marcet-Ortega et al., 2017, Pacheco et al., 2015, Rinaldi et al., 2017). The second operates in the absence of persistent DNA damage and responds instead to asynapsis (Di Giacomo et al., 2005, Wojtasz et al., 2012). Homologs asynapsed at pachynema undergo meiotic silencing, a megabase-scale chromatin remodeling process that inactivates hundreds of genes (Inagaki et al., 2010). Evidence suggests that meiotic silencing eliminates germ cells with asynapsis by depriving them of critical gene products (Cloutier et al., 2015).

In addition to its surveillance role, meiotic silencing is responsible for the inactivation of the asynapsed XY chromosome regions during male meiosis. This process, meiotic sex chromosome inactivation (MSCI), affects most or all XY genes and results in the formation of the condensed XY body (Yan and McCarrey, 2009, McKee and Handel, 1993, Solari, 1964). Disturbances in MSCI lead to misexpression of toxic sex genes and midpachytene germ cell failure (Royo et al., 2010). MSCI defects have been invoked as a cause of meiotic infertility in intersubspecific hybrids (Bhattacharyya et al., 2013, Campbell et al., 2013) and mice exhibiting chromosome abnormalities, including X-autosome translocations (Homolka et al., 2007) and Double Y syndrome (Royo et al., 2010).

**Figure 1:**
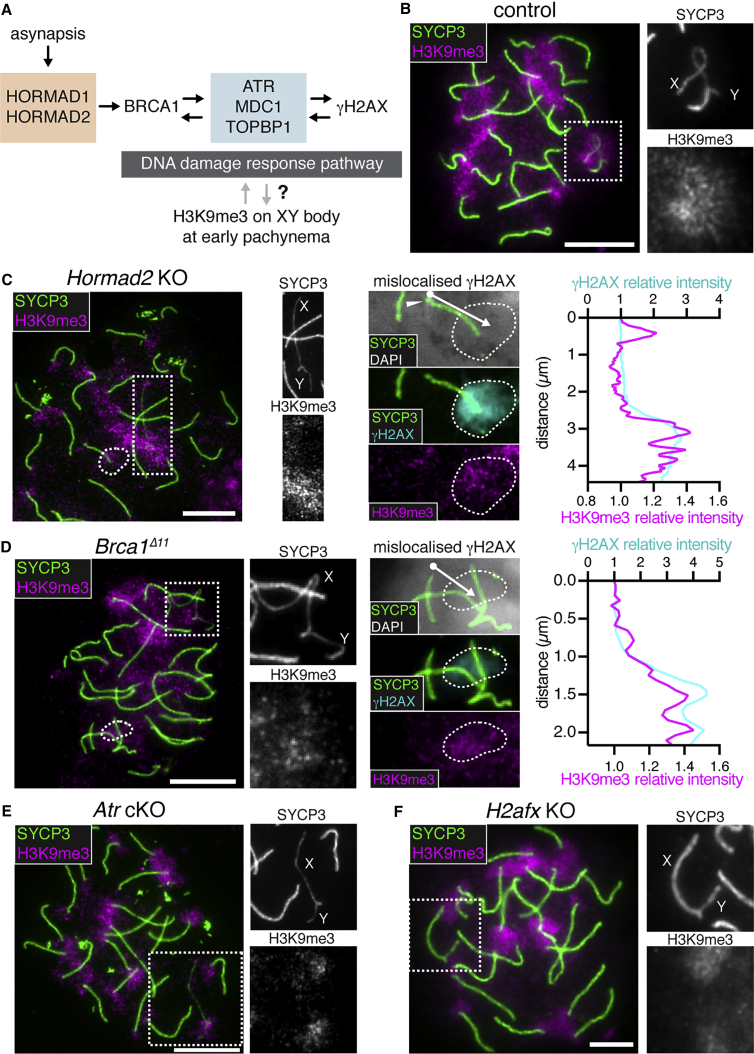
DNA Damage Response Factors Direct H3K9me3 Acquisition on the X Chromosome (A) Schematic of the MSCI pathway. (B–F) Early pachytene spermatocytes from control (B, n = 38 cells), *Hormad2* KO (C, n = 32 cells), *Brca1*^*Δ11*^ (D, n = 17 cells), *Atr* cKO (E, n = 20 cells), and *H2afx* KO (F, n = 34 cells) immunostained for SYCP3 (green) and H3K9me3 (magenta). Dashed rectangles highlight XY pair, which is magnified in right panels. Far right panels (C and D) show magnified images of mislocalized γH2AX (cyan, dashed circle) and plot profiles of relative fluorescence intensity of γH2AX (cyan) and H3K9me3 (magenta). Arrows show lines used for plot profile analysis. Arrowhead shows DAPI-dense pericentric heterochromatin. 8-week-old mice were used for analyses. Scale bars: 5 μm. See also Figure S1.

Mechanistically, meiotic silencing initiates from recombinational DNA double-strand breaks (DSBs) that are located within asynapsed chromosome axes (ElInati et al., 2017, Carofiglio et al., 2013, Schoenmakers et al., 2008). Supported by SYCP3 and HORMAD1/2, BRCA1-A complex components and ATR localize to these DSBs and thereafter spread along the full length of asynapsed chromosome axes (Lu et al., 2013, Royo et al., 2013, Wojtasz et al., 2012, Daniel et al., 2011, Kouznetsova et al., 2009, Sciurano et al., 2007, Turner et al., 2004, Xu et al., 2003). Subsequently, facilitated by MDC1 (Ichijima et al., 2011) and TOPBP1 (ElInati et al., 2017), ATR spreads into chromatin loops, catalyzing serine-139 phosphorylation of histone H2AX (Cloutier et al., 2015, Fernandez-Capetillo et al., 2003) (γH2AX; Figure 1A).

While it is clear that the DNA damage response (DDR) network has a critical role in meiotic silencing, how it ultimately induces the inactive chromatin state is not known. One possibility is that DDR components direct acquisition of canonical repressive histone modifications at asynapsed chromosomes. Based on their localization to the XY body, a number of candidate modifications have been identified, but none has yet been shown to be essential for the initiation of silencing (van der Heijden et al., 2007, Namekawa et al., 2006, Khalil et al., 2004, Baarends et al., 1999). Among these candidates, we were drawn to Histone H3 lysine 9 (H3K9) methylation. H3K9 monomethylation does not exhibit preferential XY localization (Kato et al., 2015), and H3K9 dimethylation appears on the XY pair at the pachytene-to-diplotene transition, too late for a role in MSCI initiation (Namekawa et al., 2006, Khalil et al., 2004). However, H3K9 trimethylation (H3K9me3) is observed on the XY body at early pachynema (Kato et al., 2015, Page et al., 2012, van der Heijden et al., 2007, Khalil et al., 2004) and is downstream of the DDR in mitotic cells (Ayrapetov et al., 2014, Sun et al., 2009).

Here, we show that H3K9me3 enrichment on the asynapsed X chromosome is directed by DDR factors. Using a conditional knockout (cKO) approach, we demonstrate that H3K9me3 acquisition is dependent on the H3K9 methyltransferase (MTase) SETDB1 and that *Setdb1* deletion disrupts XY body formation and XY gene silencing. We find that SETDB1 recruitment to the XY pair is dependent on H2AX and identify TRIM28 as a candidate bridging factor. Thus, our results identify SETDB1 as the link between the DDR network and meiotic chromosome silencing.

## Results

### DDR Factors Direct H3K9me3 Acquisition on the X Chromosome at Pachynema

Using SYCP3 immunostaining to label chromosome axial elements, we first confirmed previous reports (Kato et al., 2015, Page et al., 2012, van der Heijden et al., 2007, Khalil et al., 2004) that H3K9me3 is enriched on the XY bivalent, as well as on pericentric heterochromatin, at early pachynema (Figure 1B). H3K9me3 was not observed along chromosome axes or at persistent meiotic DSB sites (Figure S1A). We then investigated genetic interactions between XY-associated H3K9me3 and meiotic silencing components. We assayed sex chromosome H3K9me3 patterns in *Hormad2* knockouts (KOs) (Wojtasz et al., 2012) (Figure 1C), *Brca1* exon 11 deletion mutants (Turner et al., 2004, Xu et al., 2003) (*Brca1*^*Δ11*^; Figure 1D), *Atr* cKOs (Widger et al., 2018) (Figure 1E), and *H2afx* KOs (Celeste et al., 2002) (Figure 1F), all of which exhibit defective MSCI and resulting germ cell failure at midpachynema. We restricted our analysis at this stage to the X chromosome because the heterochromatic Y chromosome is constitutively positive for H3K9me3, even in spermatogonia (Baumann et al., 2008) (Figure S1B). Although preserved at pericentric heterochromatin, H3K9me3 was not observed on the X chromosome in these mutants. H3K9me3 enrichment to the X chromosome is therefore downstream of DDR factors.

Examination of *Hormad2* KOs and *Brca1*^*Δ11*^ mutants revealed further information on the relationship between DDR factors and H3K9me3. As well as defective MSCI, these mutants exhibit another phenotype: ATR normally destined for the X chromosome mislocalizes to synapsed autosomes, where it induces ectopic domains of γH2AX (Wojtasz et al., 2012, Turner et al., 2004). We found that in *Hormad2* KOs and *Brca1*^*Δ11*^ mutants, these ectopic γH2AX domains were also enriched for H3K9me3 (Figures 1C, 1D, and S1C). These findings suggest a close spatial relationship between γH2AX and H3K9me3 acquisition.

### Meiotic *Setdb1* Deletion Causes Midpachytene Apoptosis

**Figure 2:**
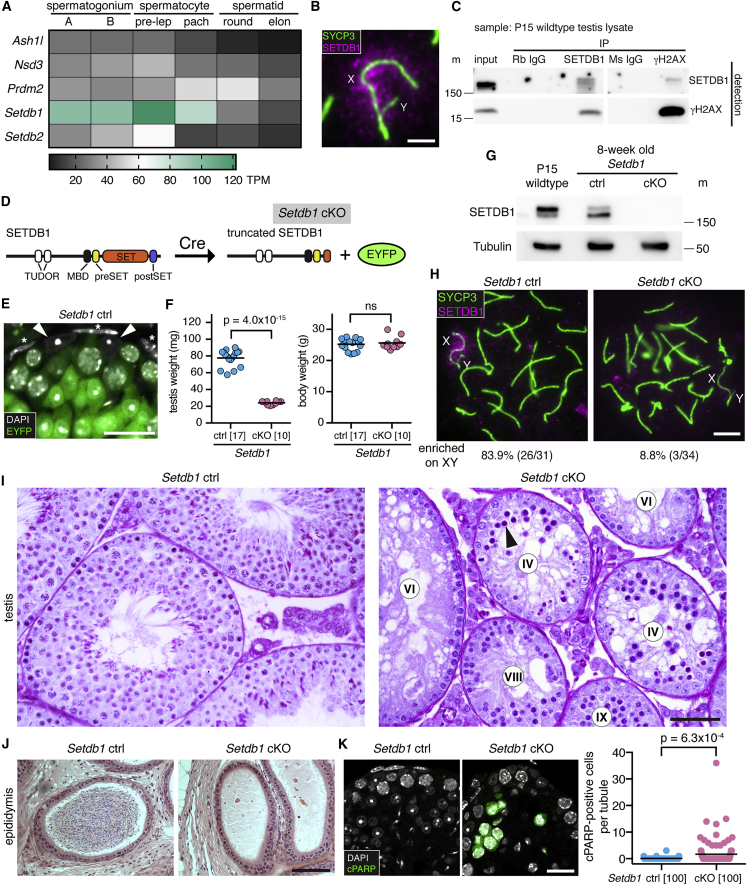
*Setdb1* Deletion Causes Midpachytene Apoptosis (A) H3K9me3 MTase expression in male germ cells by RNA-seq. A: typeA; B: type B; pre-lep: pre-leptonema; pach: pachynema; elon: elongated; TPM: transcripts per million. (B) *Setdb1* control XY bivalent immunostained for SYCP3 (green) and SETDB1 (magenta). See quantitation in (H). Scale bar: 2 μm. (C) Western blot of input and immunoprecipitated samples using P15 wild-type testis lysate treated with nuclease. Rb: rabbit; Ms: mouse; m: size marker. Expected size: 180 kDa (SETDB1), 17 kDa (γH2AX). (D) Schematic of SETDB1 domain structure before and after Cre recombination. *Setdb1* cKO also expresses EYFP from *Gt(ROSA)26Sor* Cre reporter locus. (E) Testis section of *Setdb1* control immunostained for EYFP (green, stained with GFP antibody). Note that EYFP is negative in Sertoli cells (arrowheads) and peritubular myoid cells (asterisks). Scale bar: 20 μm. (F) Testis and body weights in *Setdb1* control and cKO mice. Number of mice analyzed in brackets. ns: not significant. p value calculated using unpaired t test. (G) Testis SETDB1 western blot of *Setdb1* control and cKO. Tubulin was used as a loading control. 50 μg of protein per lane was loaded. m: size marker. Expected size: 180 kDa (SETDB1), 50 kDa (Tubulin). SETDB1 antibody used recognizes two SETDB1 bands, of which the upper band is considered to be ubiquitinated SETDB1 (Ishimoto et al., 2016). (H) Early pachytene *Setdb1* controls and *Setdb1* cKOs immunostained for SYCP3 (green) and SETDB1 (magenta). Percentages of cells positive for SETDB1 signals on XY are shown below panels. (I and J) Histology of *Setdb1* control and cKO testes (I, periodic acid-Schiff staining) and epididymides (J, hematoxylin and eosin staining). Arrowhead: apoptotic pachytene cell. Number labels show tubule stages. Scale bars: 50 μm (I), 100 μm (J). (K) Testis sections of *Setdb1* control and cKO immunostained for cleaved PARP (green). Chart shows number of cells positive for cleaved PARP per tubule. Number of tubules analyzed in brackets. p value calculated using unpaired t test. Scale bar: 20 μm. 8-week-old mice were used for analyses except for P15 sample in (C) and (G). See also Figure S2.

The enzyme responsible for meiotic X chromosome-associated H3K9me3 is unknown. H3K9me3-catalyzing MTases SUV39H1 and its paralog SUV39H2 are dispensable for XY body-associated H3K9 methylation (Peters et al., 2001). To identify alternative H3K9me3 MTase candidates, we analyzed published RNA sequencing (RNA-seq) data from mouse spermatogenic subpopulations (Gan et al., 2013). *Setdb1* caught our attention because its expression exceeded that of other candidate H3K9me3 MTases during pachynema (Figure 2A). SETDB1 localized to the XY pair at early pachynema (Figure 2B). Furthermore, testis immunoprecipitation (IP) followed by western blotting revealed that SEDTB1 and γH2AX form a complex by either direct or indirect interaction (Figure 2C). In mice, constitutive deletion of *Setdb1* causes embryonic lethality (Dodge et al., 2004), and conditional ablation in early germ cells causes spermatogenic arrest before meiosis (An et al., 2014, Liu et al., 2014). We therefore sought to delete *Setdb1* later in spermatogenesis, prior to pachynema.

To achieve meiotic *Setdb1* deletion, we generated *Setdb1* cKO *Setdb1*^flox/−^ male mice carrying a *Ngn3-Cre* transgene (Schonhoff et al., 2004). The *Setdb1* cKO mutation deletes the core amino acids in the catalytic SET domain (Matsui et al., 2010) (Figure 2D). *Ngn3-Cre* is expressed in the gastrointestinal tract and pancreas, as well as in male germ cells from post-natal day (P) 7, and has been used to efficiently deplete *Topbp1*, *Atr*, *and Mov10l1* during meiosis (ElInati et al., 2017, Widger et al., 2018, Zheng and Wang, 2012). We confirmed using a ROSA26-EYFP Cre reporter that *Ngn3-Cre* is active in germ cells from the spermatogonial stage and is not active in somatic cells of the testis (Figure 2E). This Cre transgene was superior to *Stra8-Cre* (Sadate-Ngatchou et al., 2008) at achieving efficient meiotic SETDB1 depletion (Figures S2A–S2C). The mean testis weight in 8-week-old *Setdb1* cKO males was reduced relative to *Setdb1* control (*Setdb1*^*flox/+*^; *Ngn3-Cre*) males, while the mean body weight was unaffected (Figure 2F). SETDB1 protein levels were reduced in *Setdb1* cKO testes (Figure 2G), which was confirmed by immunofluorescence analysis of nuclear spreads (Figure 2H). Although the conditional mutation may have been expected to generate a truncated SETDB1, no such protein was identified by western blotting in *Setdb1* cKO testis (Figure S2D). Germ cell progression in *Setdb1* cKO males was unaffected up to stage IV, corresponding to midpachynema of meiosis. At this point, there was a complete block in germ cell development (Figure 2I). As a result, later germ cell types were absent, and sperm were not present in the cauda epididymides (Figure 2J). *Setdb1* cKO tubule sections contained elevated numbers of cleaved-PARP stained spermatocytes (Figure 2K). Germ cell loss in *Setdb1* cKOs therefore occurs via apoptosis.

### Minor Effects of *Setdb1* Deletion on Meiotic Recombination and Synapsis

Midpachytene germ cell failure can be caused by defects in homologous recombination, synapsis, or MSCI. We first established whether homologous recombination was affected in *Setdb1* cKOs. For this purpose, we counted foci of the meiotic DSB markers RPA2 and RAD51 at leptonema and early pachynema. Relative to controls, at leptonema in *Setdb1* cKOs, the mean RPA2 count was unchanged, and the mean RAD51 count was marginally decreased (Figures S3A and S3B). At early pachynema in *Setdb1* cKOs, the mean RPA2 count was slightly higher while the mean RAD51 count was unaffected (Figures S3C and S3D). We also found that at early pachynema, RPA2 and RAD51 counts on the X chromosome and at the PAR were similar between *Setdb1* cKOs and controls (Figures S3E and S3F). Thus, *Setdb1* deletion had a minimal effect on the abundance of these recombination markers.

**Figure 3:**
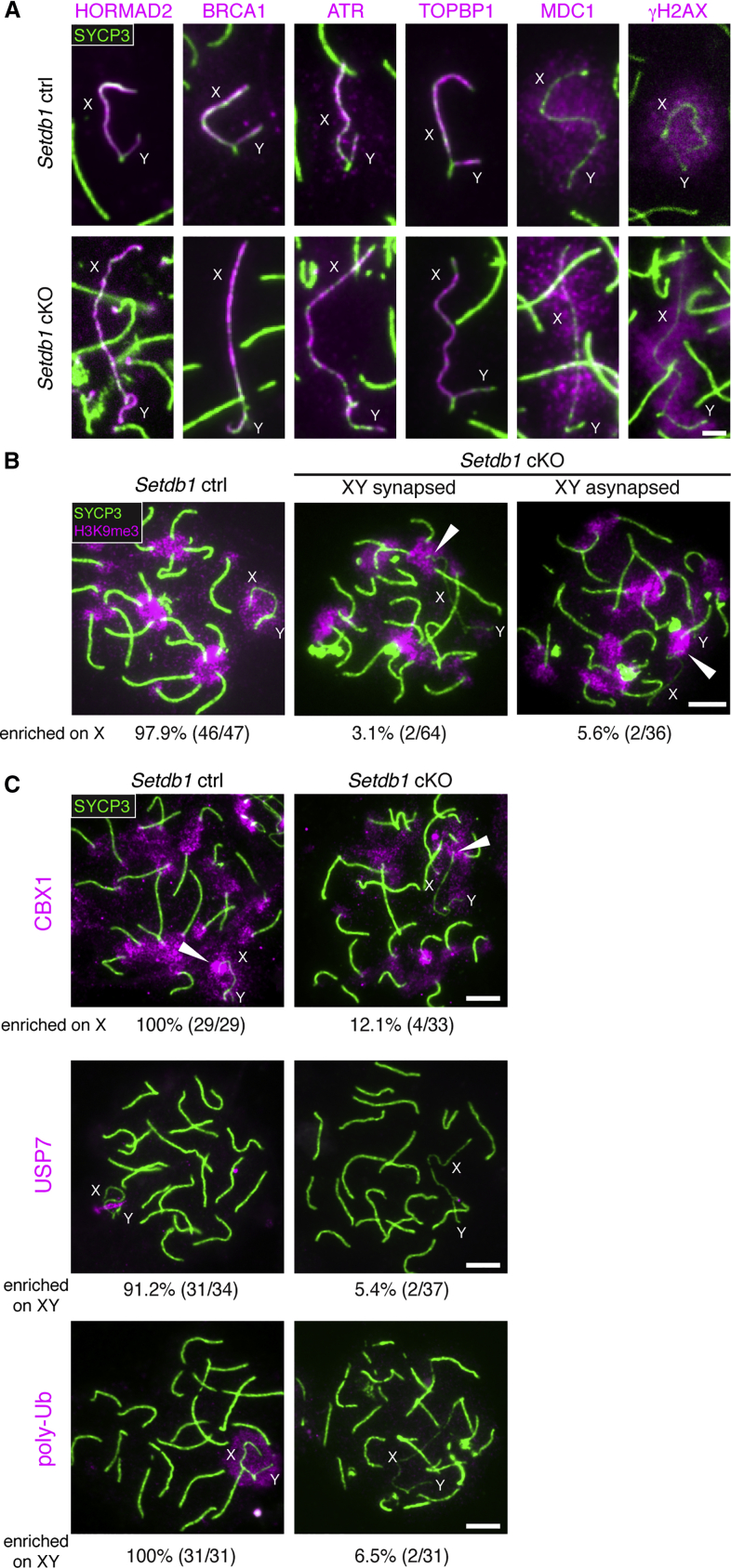
SETDB1 Is Required for Epigenetic Remodeling of the XY Pair (A) Early pachytene *Setdb1* control and cKO XY bivalents immunostained for SYCP3 (green) and silencing factors (magenta; n ≥ 30 cells for each factor). Scale bar: 2 μm. (B) Early pachytene *Setdb1* controls and cKOs immunostained for SYCP3 (green) and H3K9me3 (magenta). Arrowheads: H3K9me3 on pericentric heterochromatin of X. Percentages of cells positive for H3K9me3 signals on non-pericentric X are shown below panels. Scale bar: 5 μm. (C) Early (CBX1) and mid (USP7 and poly-Ub) pachytene *Setdb1* controls and cKOs immunostained for SYCP3 (green) and indicated factors (magenta). Arrowheads: CBX1 on pericentric heterochromatin of X. Percentages of cells positive for signals on non-pericentric X (CBX1) or XY (USP7 and poly-Ub) are shown below panels. Scale bar: 5 μm. 8-week-old mice were used for analyses. See also Figures S3 and S4.

Next, we assayed synapsis in *Setdb1* cKOs. We used antibodies to centromeres and to the asynapsis marker HORMAD2, localization of which was unaffected in *Setdb1* cKOs (Figure 3A). Asynapsis was observed in 53% of *Setdb1* cKO cells but only 3% of control cells at early pachynema (Figure S4A). Despite being more common, the severity of the asynapsis phenotype in *Setdb1* cKOs was minor, most often affecting only the XY pair (Figures S4A and S4B) or a single autosomal bivalent (Figures S4A and S4C). XY and autosomal asynapsis were not always coincident in *Setdb1* cKO cells. Interestingly, autosomal asynapsis in *Setdb1* cKOs was observed exclusively at the centromeric end of the bivalent (100%; n = 27 asynapsed bivalents). In contrast, autosomal asynapsis in controls was not exclusively centromeric (77%; n = 31 asynapsed bivalents; p = 1.17 x 10^−2^; Fisher’s exact test). To assess whether the centromeric asynapsis associated with *Setdb1* deletion preferentially affected smaller autosomes, we performed DNA-fluorescence in situ hybridization (FISH) for chromosomes 1 (a large chromosome) and 19 (a small chromosome) (Figure S4D). In control spermatocytes, asynapsis of chromosome 19 was more common than asynapsis of chromosome 1 (see legend for quantitation). In the *Setdb1* cKO, the incidence of asynapsis was increased for both autosomes. However, the frequency ratio of chromosome 19 to chromosome 1 asynapsis was similar to that in the control. The role of SETDB1 in ensuring centromeric synapsis is not therefore specific for smaller autosomes.

### SETDB1 Is Required for Epigenetic Remodeling of the XY Pair

Given the mild defects in recombination and synapsis, we examined whether MSCI was perturbed in *Setdb1* cKOs. We focused first on localization of silencing factors to the XY bivalent at early pachynema. Increasing numbers of asynapsed autosomes can indirectly antagonize γH2AX accumulation on the XY pair (Mahadevaiah et al., 2008). For this reason, we initially examined cells without autosomal asynapsis. XY localization of SYCP3, HORMAD2, BRCA1, ATR, TOPBP1, MDC1, and γH2AX occurred normally in these cells, whether the sex chromosomes were synapsed (Figure 3A) or asynapsed (Figure S4E). Also in *Setdb1* cKO cells with autosomal asynapsis, XY γH2AX localization was unimpaired (Figure S4F). The magnitude of asynapsis in this mutant thus falls below that required to antagonize XY γH2AX accumulation (Mahadevaiah et al., 2008). Importantly, however, while in *Setdb1* cKOs, H3K9me3 localization to pericentric heterochromatin and to the Y chromosome was unaffected, localization to the asynapsed X chromosome did not occur (Figure 3B).

We next assessed the impact of *Setdb1* deletion on other XY chromatin associated factors. CBX1, also known as heterochromatin protein 1 (HP1) beta, directly binds to H3K9me3 (Bannister et al., 2001) and facilitates spreading of this mark during heterochromatin formation (Mozzetta et al., 2015). In controls, CBX1 was observed on the XY body not only at diplonema, as previously reported (Metzler-Guillemain et al., 2003, Turner et al., 2001, Motzkus et al., 1999), but also at early pachynema. As observed for H3K9me3, in *Setdb1* cKOs, CBX1 was preserved at pericentric heterochromatin and at the Y chromosome but was lost at the asynapsed X chromosome (Figure 3C). Pathways regulating later stages of XY chromatin remodeling were also perturbed in this mutant. H2A lysine-119 deubiquitylating enzyme USP7, which is recruited by SCML2 (Hasegawa et al., 2015, Luo et al., 2015), was not observed on the XY pair (Figure 3C). Similarly, RNF8-dependent polyubiquitylation (poly-Ub), which is required for reactivation of sex chromosomes after meiosis (Sin et al., 2012), was absent (Figure 3C). Thus, SETDB1 acts downstream of the DDR pathway in XY chromatin remodeling by catalyzing H3K9me3.

### SETDB1 Is Required for Condensation of the XY Pair

**Figure 4:**
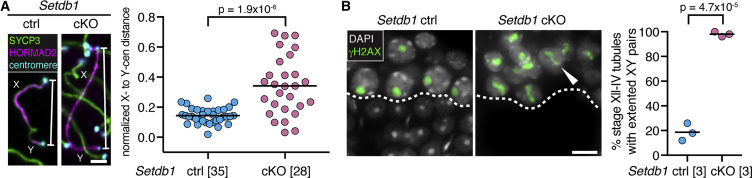
SETDB1 Is Required for Condensation of the XY Pair (A) Immunostaining and quantification of early pachytene X-Y centromere distance in *Setdb1* controls and cKOs (SYCP3: green; HORMAD2: magenta; centromeres: cyan). Number of cells analyzed in brackets. p value calculated using Mann-Whitney test. Scale bar: 2 μm. (B) Immunostaining and quantification of XY condensation in *Setdb1* control and cKO testis sections (γH2AX: green; DAPI: white). Dashed line: boundary between spermatocytes and spermatids. In *Setdb1* cKOs, spermatids are absent as a result of stage lV block. Arrowhead: example of extended XY pair. Chart represents percentage of stage Xll–lV tubules with extended XY pairs (50 tubules per mouse analyzed; three mice per genotype). p value calculated using unpaired t test. Scale bar: 10 μm. 8-week-old mice were used for analyses.

During MSCI, the extended XY pair condenses to form the XY body. We noted that at early pachynema, *Setdb1* cKOs exhibited persistently extended XY bivalents. To quantitate this phenotype, we immunolabeled sex chromosomes for SYCP3, HORMAD2, and centromeres and used an established approach to measure the mean distance between the X and Y centromeres (Ichijima et al., 2011). In *Setdb1* cKO early pachytene cells, the mean X-to-Y centromere distance was 2-fold higher than that in controls (Figure 4A), indicating that XY body formation in this mutant was defective. To confirm this phenotype, we analyzed the condensation state of γH2AX-immunolabeled XY pairs in stage-matched *Setdb1* cKO and control testis sections. In controls, XY pairs were extended during stage XII (late zygonema) and condensed during stages I to IV (early-to-mid pachynema). However, in *Setdb1* cKOs, XY pairs were extended during stage XII and thereafter remained extended from stage I to stage IV, when germ cell elimination takes place (Figure 4B). SETDB1 is therefore essential for sex chromosome condensation.

### SETDB1 Is Required for XY Silencing at Pachynema

Condensation of the XY pair is associated with sex-gene silencing at pachynema (Inagaki et al., 2010, Yan and McCarrey, 2009). To establish effects of *Setdb1* deletion on XY gene expression, we performed RNA-seq on fluorescence-activated cell sorting (FACS)-purified (Bastos et al., 2005) early-to-mid pachytene cells from *Setdb1* cKOs and wild-type C57BL6/J males. An initial analysis of published RNA-seq data (Gan et al., 2013) showed that the mean X- and Y-gene transcripts per million (TPM) in purified pachytene cells are lower than in purified spermatogonia and preleptotene cells (Figure S5A). The mean X- and Y-gene TPMs in our wild-type purified pachytene population was similar to those observed in this published study. However, in purified *Setdb1* cKO pachytene cells, the mean X- and Y-gene TPMs were elevated (Figure S5A), suggesting that XY silencing was defective.

**Figure 5:**
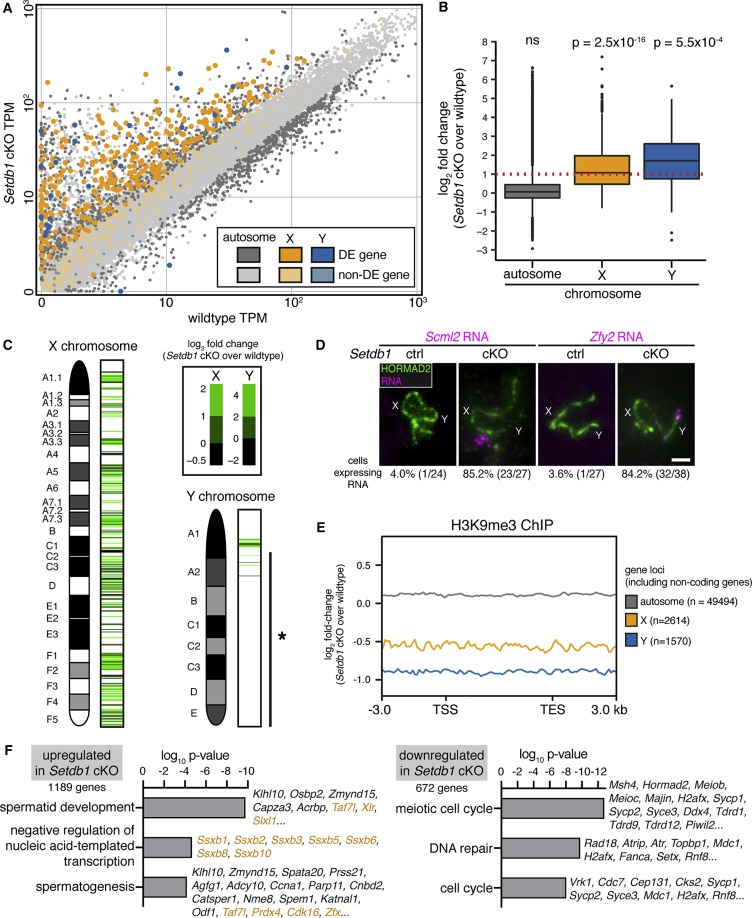
Defective MSCI in *Setdb1* cKO Pachytene Spermatocytes (A) Comparison of gene expression levels between wild-type ad *Setdb1* cKO pachytene cells. DE: differentially expressed. DE genes: autosome (dark gray); X (dark orange); Y (dark blue). non-DE genes: autosome (light gray); X (light orange); Y (light blue). TPM: transcripts per million. (B) Boxplot represents gene expression log2-fold change of *Setdb1* cKO relative to wild-type. Box: 25th/75th percentiles. Line on box: median. Whisker: 1.5 times the interquartile range from the 25th/75th percentiles. Red dashed line: 2-fold change. ns: not significant. p value calculated using Welch’s t test. (C) Heatmaps representing gene expression log2-fold change of *Setdb1* cKO relative to wild-type across different chromosomal locations. Asterisk: long arm of the Y chromosome, which is occupied by multicopy genes (*Ssty1*, *Ssty2*, *Sly*, *Asty*). Note that the software used for these heatmaps excludes multi-mapped X and Y genes. (D) X-linked *Scml2* and Y-linked *Zfy2* RNA-FISH (magenta) of *Setdb1* controls and cKOs immunostained for HORMAD2 (green). Percentages of cells positive for RNA signals are shown below panels. Scale bar: 2 μm. (E) H3K9me3 occupancy log2-fold change of *Setdb1* cKO relative to wild-type. Gray: autosomal genes; orange: X genes; blue: Y genes. Analysis includes all coding and noncoding genes. TSS: transcription start site. TES: transcription end site. (F) The top three ontology terms enriched in upregulated and downregulated genes in *Setdb1* cKO cells. Example genes are listed on the right. Orange: X-linked genes. See also Figures S5–S7 and Tables S1 and S2.

We next performed differential expression analysis. 63% (304/784) of X-protein-coding and 38% (29/105) of Y-protein-coding genes were differentially expressed between *Setdb1* cKOs and wild-type pachytene cells. Furthermore, almost all of these XY genes were expressed more highly in the *Setdb1* cKOs than in the wild-types (99.7% of X- and 93.1% of Y-encoded differentially expressed genes; Figure 5A and Table S1). Upregulated Y genes included *Zfy1* and *Zfy2*, misexpression of which causes midpachytene germ cell elimination (Royo et al., 2010). As a result of this skewed XY expression change, median expression from the X and Y chromosomes in *Setdb1* cKOs was increased 3.3- and 3.7-fold, respectively, relative to wild-types (Figure 5B). In contrast, only 9% of autosomal genes were differentially expressed, and of these genes, 56.2% were upregulated and 43.7% downregulated in *Setdb1* cKOs compared to wild-types (n = 1,528 genes). Consequently, median expression from the autosomes was unchanged (Figure 5B). An elevation in median X and Y expression was not observed in published RNA-seq datasets derived from *Setdb1* cKO embryonic stem cells (ESCs) or primordial germ cells (PGCs) and was thus a pachytene-specific effect (Figures S5B and S5C). The X-to-autosome (X:A) ratio in *Setdb1* cKO pachytene cells was elevated compared to that in wild-types (Figure S5D), consistent with preferential overexpression of X versus autosomal genes in the former genotype. Genes upregulated in the *Setdb1* cKO were found across the length of the X chromosome (Figure 5C; see legend for discussion of Y chromosome). Thus, *Setdb1* deletion caused upregulation of sex-linked genes in pachytene cells but not in ESCs or PGCs.

We complemented our transcriptomic analysis with RNA-FISH for the X gene *Scml2* and the Y gene *Zfy2*. These genes are silenced at pachynema in control males but not in MSCI mutants (ElInati et al., 2017, Royo et al., 2010, Bhattacharyya et al., 2013, Royo et al., 2013). Following RNA-FISH, early pachytene cells were identified using HORMAD2 immunostaining, as described previously (Cloutier et al., 2015, Cloutier et al., 2016). Both *Scml2* and *Zfy2* were misexpressed at early pachynema in *Setdb1* cKOs, and the proportion of cells exhibiting *Scml2* and *Zfy2* RNA-FISH signals was similar to that observed in established MSCI mutants (ElInati et al., 2017, Wojtasz et al., 2012) (Figure 5D). Thus, transcriptomic and RNA-FISH analyses demonstrate that SETDB1 is critical for MSCI.

Based on our immunostaining analysis (Figure 3B), we hypothesized that SETDB1 mediates silencing by directing H3K9me3 acquisition at X genes. We performed ultra-low-input native chromatin immunoprecipitation sequencing (ChIP-seq) (Brind’Amour et al., 2015) to compare H3K9me3 occupancy at all genes (coding and non-coding) in early-to-mid pachytene *Setdb1* cKOs and wild-type cells. H3K9me3 levels at autosomal genes were similar between these two genotypes. However, H3K9me3 levels at X and Y genes were reduced in the *Setdb1* cKO relative to the wild-type (Figure 5E). The reduction in H3K9me3 was observed both at XY genes upregulated in the *Setdb1* cKO and at XY genes that were not upregulated (Figure S6). These findings indicate that in the *Setdb1* cKO reduction of XY H3K9me3 occurs at a chromosomal level.

We examined putative functions of genes upregulated upon depletion of the repressive H3K9me3 mark (Figure 5F and Table S2). Genes upregulated in *Setdb1* cKOs relative to wild-types were enriched most highly for the Gene Ontology (GO) category “spermatid development.” This finding was surprising because spermatids were absent in *Setdb1* cKOs (Figures 2I and 2J). The “spermatid development” category included genes mapping to the X chromosome, where spermatid genes are abundant (Mueller et al., 2008), as well as autosomal genes. Essential functions in spermiogenesis have been described for several of these X genes, e.g., *Taf7l* (Cheng et al., 2007) and *Slxl1* (Cocquet et al., 2010), and autosomal genes, e.g., *Klhl10* (Yan et al., 2004), *Osbp2* (Udagawa et al., 2014), *Zmynd15* (Clark et al., 2004), *Capza3* (Geyer et al., 2009), *Acrbp* (Kanemori et al., 2016), and *Six5* (Sarkar et al., 2004). We also performed GO analysis on genes downregulated in *Setdb1* cKOs relative to wild-types (Figure 5F). The most significantly enriched categories featured genes functioning in meiosis. These findings indicate that *Setdb1* regulates the timely expression of spermatogenesis genes, promoting expression of meiosis genes and preventing premature expression of spermatid genes.

### A Subset of ERVs Is Upregulated in the *Setdb1 cKO*

SETDB1 silences endogenous retroviruses (ERVs) in somatic cells (Kato et al., 2018) and PGCs (Liu et al., 2014). Using our RNA-seq data, we addressed whether SETDB1 performs a similar function in pachytene cells. 5% (38/836) of ERVs were upregulated in the *Setdb1* cKO relative to the wild-type (Figure S7A and Table S3). Upregulated ERVs were derived from multiple families, with the most highly overexpressed ERV being MMERVK10C-int. A similar proportion of ERVs (43/836) was downregulated in the *Setdb1* cKO relative to the wild-type. In the *Setdb1* cKO, upregulated ERVs showed a more marked decrease in H3K9me3 occupancy than downregulated ERVs or non-differentially expressed ERVs (Figures S7B and S7C). In conclusion, the effect of *Setdb1* deletion on ERV silencing in pachytene cells is milder than observed in other contexts (Karimi et al., 2011, Kato et al., 2018, Liu et al., 2014, Matsui et al., 2010).

### TRIM28 Is a Candidate DDR-SETDB1 Bridging Factor in MSCI

**Figure 6:**
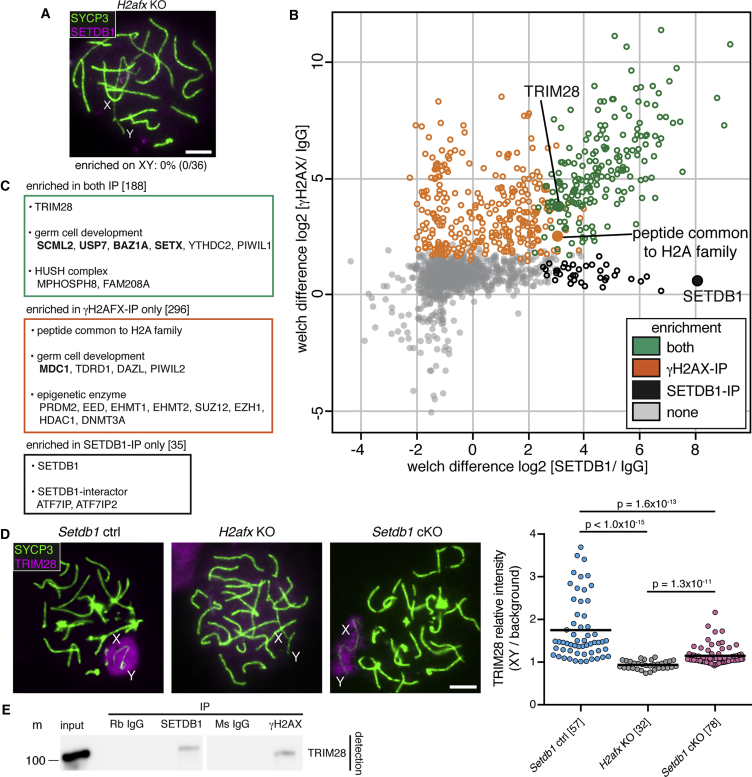
Genetic and Protein Interaction of SETDB1 with γH2AX and TRIM28 (A) Early pachytene *H2afx* KOs immunostained for SYCP3 (green) and SETDB1 (magenta). Percentages of cells positive for SETDB1 signals on XY are shown below panels. (B) IP-MS analysis to identify SETDB1- and γH2AX-interactors in testes. Axis values show LFQ intensity log2-fold change of SETDB1-IP (X axis) or γH2AX-IP (Y axis) relative to control immunoglobulin G (IgG)-IP. Green: enriched in both γH2AX- and SETDB1-IP. Orange: enriched only in γH2AX-IP. Black: enriched only in SETDB1-IP. Gray: non-enriched. Detection of H2AX is limited due to sequence similarity among H2A family proteins. (C) Examples of proteins enriched in γH2AX- and/or SETDB1-IP. Number of proteins enriched in brackets. Bold: known MSCI factors. (D) Early pachytene *Setdb1* controls, *H2afx* KOs, and *Setdb1* cKOs immunostained for SYCP3 (green) and TRIM28 (magenta). Chart shows relative intensity of TRIM28 on XY relative to non-XY region in nucleus. Number of cells analyzed in brackets. p value calculated using Mann-Whitney test. (E) Western blot of input and immunoprecipitated samples using P15 wild-type testis lysate treated with nuclease. Rb: rabbit; Ms: mouse; m: size marker. Expected TRIM28 size: 110 kDa. 8-week-old mice were used in (A) and (D); P15 mice were used in (B) and (E). Scale bars: 5 μm. See also Figure S7D and Table S4.

Our findings showed that SETDB1 acts downstream of the DDR network. Consistent with this conclusion, SETDB1 localization to the XY bivalent did not occur in *H2afx* KO pachytene cells (Figure 6A). However, the mechanism underlying sex chromosome SETDB1 recruitment was unclear. TRIM28, hnRNP K, and Krüppel-associated box zinc-finger proteins (KRAB-ZFPs) recruit SETDB1 in other contexts (Ecco et al., 2017, Iyengar and Farnham, 2011, Thompson et al., 2015), and TRIM28 is also involved in the DDR (White et al., 2006). To assess which of these cofactors could be important in XY-SETDB1 recruitment, we performed testis IP-mass spectrometry (MS) on P15 wild-type testis using both γH2AX and SETDB1 as bait (Figure 6B and Table S4). Proteins significantly enriched in both the γH2AX and SETDB1 IP-MS experiments included TRIM28 and hnRNP K but not KRAB-ZFPs. Both IP-MS experiments also pulled down known XY body-associated proteins (e.g., SCML2, USP7) (Hasegawa et al., 2015, Luo et al., 2015), as well as components of the HUSH complex, which promote SETDB1-mediated H3K9me3 spreading (Tchasovnikarova et al., 2015) (Figure 6C). Proteins observed only in the γH2AX IP-MS included MDC1 and several epigenetic enzymes, while those unique to the SETDB1 IP-MS included the SETDB1-stabilizing factors ATF7IP and ATF7IP2 (Ichimura et al., 2005, Timms et al., 2016) (Figure 6C). IP-MS can thus identify additional candidate XY-silencing factors.

To further assess their candidacy, we analyzed TRIM28 and hnRNP K immunostaining in early pachytene cells. hnRNP K was present throughout autosomal chromatin but was notably excluded from the XY pair (Figure S7D). However, TRIM28 localized to the sex chromosomes, further supporting a role for this protein in linking the DDR to SETDB1 (Figure 6D). Testis IP followed by western blotting confirmed that both SETDB1 and γH2AX interact with TRIM28 (Figure 6E). In *H2afx* KO pachytene cells, localization of TRIM28 to the XY pair was abolished (Figure 6D), placing TRIM28 downstream of the DDR network in MSCI. TRIM28 was observed on the XY pair in the *Setdb1* cKO (Figure 6D). However, the staining intensity of XY-associated TRIM28 was lower than that in the control (Figure 6D). Thus, while TRIM28 is upstream of SETDB1 in MSCI, SETDB1 may facilitate TRIM28 amplification on the sex chromosomes (see Discussion). Overall, our findings support a role for TRIM28 in bridging the DDR to SETDB1.

## Discussion

A fundamental role for the DDR network in initiating meiotic silencing is well established. DDR proteins collaborate to induce phosphorylation of H2AX on asynapsed chromosomes. However, whether resulting γH2AX is sufficient to silence transcription has been unclear. Here, we show that the repressive histone mark H3K9me3, catalyzed by SETDB1, is an additional critical step downstream of γH2AX in MSCI, driving sex chromosome condensation and XY gene silencing. The midpachytene apoptosis in the *Setdb1* cKO could be attributed to toxic sex-gene expression (Royo et al., 2010, Royo et al., 2015) and/or de-repression of a subset of ERVs. SETDB1 represses ERVs in various contexts (Collins et al., 2015, Karimi et al., 2011, Kato et al., 2018, Liu et al., 2014, Matsui et al., 2010) and has developmental functions that include oogenesis (Eymery et al., 2016, Kim et al., 2016), early development (Dodge et al., 2004), and neurogenesis (Tan et al., 2012). Our findings reveal a distinct role in male meiosis, where it links the meiotic DDR network to H3K9me3.

**Figure 7:**
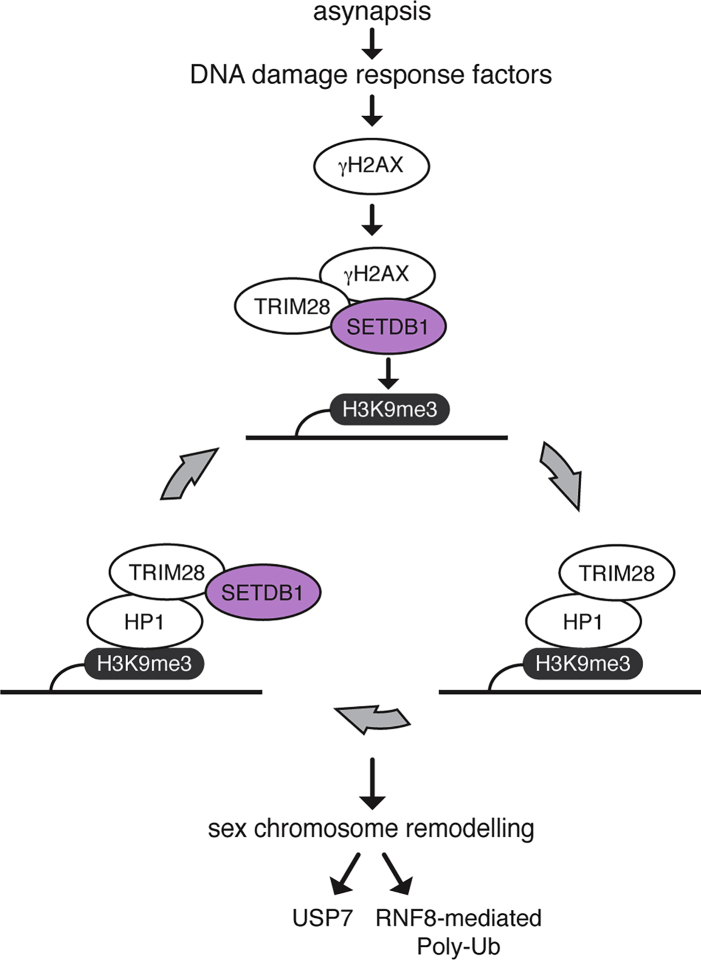
Model Explaining the Role of SETDB1 in MSCI Asynapsis sensors and DNA damage response factors are recruited to XY pair, resulting in γH2AX accumulation. γH2AX recruits TRIM28 and SETDB1, which mediates H3K9me3 acquisition. HP1 (CBX1, CBX3, CBX5)-TRIM28 complex binds to H3K9me3 and recruits more SETDB1, facilitating the repressive chromatin state. USP7 and RNF8-mediated poly-Ub pathways act downstream of H3K9me3 for further sex chromosome remodeling.

Our data suggest that SETDB1 interacts with the DDR network via TRIM28 (Figure 7). Localization of TRIM28 to the XY pair requires H2AX but not SETDB1. However, in the absence of SETDB1, levels of sex chromosome-associated TRIM28 are reduced relative to the control. We therefore propose that SETDB1 ensures optimal XY-associated TRIM28-enrichment. In mitotic cells, TRIM28 recruits SETDB1, with resulting H3K9me3 acting as a binding site for HP1 proteins (CBX1, CBX3, CBX5) (Bannister et al., 2001, Matsui et al., 2010, Rowe et al., 2010, Schultz et al., 2002). Because HP1 can directly bind to TRIM28 (Ryan et al., 1999), a rolling cycle amplification could then be initiated, recruiting more SETDB1. Similar positive-feedback systems are commonplace in MSCI. For example, accrual of BRCA1 and ATR at chromosome axes is interdependent (Royo et al., 2013, Turner et al., 2004), as is enrichment of ATR, MDC1, and γH2AX at chromatin loops (Royo et al., 2013).

*Setdb1* deletion disrupts XY localization of CBX1 at early pachynema. CBX1 and other HP1 family proteins form dimers bridging two H3K9me3 nucleosomes in heterochromatin (Machida et al., 2018). It would be interesting to investigate the effect of meiotic perturbation of HP1 dimerization on chromosome silencing. At midpachynema in *Setdb1* cKOs, XY pairs did not acquire USP7 and poly-Ub. Previous works indicate that SCML2-USP7 and RNF8-mediated poly-Ub act independently during XY chromatin remodeling for proper spermiogenesis (Hasegawa et al., 2015, Lu et al., 2010, Sin et al., 2012). Whether CBX1 acts upstream of one or both of these pathways warrants further investigation.

We observed in *Setdb1* cKOs a mild defect in synapsis. This phenotype was observed preferentially at the PAR. Although we observed no difference between the *Setdb1* cKO and control in PAR-localization of recombination factors, SETDB1 may have a specialized role at this chromosome region. Alternatively, the PAR phenotype may reflect a more general function for SETDB1 in synapsis. PAR asynapsis is a common finding in meiotic mutants (Fernandez-Capetillo et al., 2003, Ichijima et al., 2011, Turner et al., 2004, de Vries et al., 2005, Widger et al., 2018) and may occur because this chromosome region is small in length and thus more susceptible to synapsis defects. Consistent with this hypothesis, the smallest autosome, chromosome 19, is also more commonly asynapsed than chromosome 1 in the *Setdb1* cKO. Although the mechanism by which SETDB1 regulates synapsis is unclear, our data suggest a specific role at centromeric chromosome ends. One possibility is that H3K9 methylation at pericentric regions is important for synapsis. Autosomal asynapsis, associated with defective pericentric H3K9 methylation, is also observed in mice doubly deficient for *Suv39h1* and *Suv39h2* (Peters et al., 2001). However, it is also noteworthy that autosomal centromeres, such as the XY pair, synapse later than other chromosome regions (Bisig et al., 2012, Kauppi et al., 2011). SETDB1 could therefore have a specific function in promoting synapsis at late-pairing regions.

*Setdb1* deletion at pachynema unexpectedly causes premature upregulation of genes normally expressed in spermatids and downregulation of meiotic genes. Since these autosomal genes are not direct H3K9me3 targets (Figure 5E), we speculate that their dysregulation occurs as an indirect consequence of defective MSCI. The effect of MSCI on autosomal gene expression has not been well examined. In female somatic cells, a mechanistically distinct form of X chromosome inactivation (XCI) occurs, which functions to balance somatic X-gene expression levels with that of males. Recent work demonstrates that disrupting somatic XCI causes dysregulation of transcription at the genome-wide level (Borensztein et al., 2017, Sakata et al., 2017). By analogy, we suggest that as well as silencing XY genes, MSCI regulates autosomal gene expression patterns in the mammalian germline.

In addition to mammals, meiotic silencing has been described in other organisms, including *C. elegans* (Kelly and Aramayo, 2007). In both organisms, asynapsis is the trigger for silencing, but downstream molecular events that lead to gene inactivation were thought to be distinct. For instance, meiotic silencing in *C. elegans* relies not on DDR factors, as in mammals, but instead on components of the RNA interference machinery (Kelly and Aramayo, 2007). Interestingly, a report indicates that the *Setdb1* homolog *met-2* is essential for MSCI in *C. elegans* (Checchi and Engebrecht, 2011). Our current findings are therefore significant because they identify *Setdb1* as a silencing factor conserved between these two highly diverged model organisms.

## STAR★Methods

### Key Resources Table

**Table:** 

REAGENT or RESOURCE	SOURCE	IDENTIFIER
**Antibodies**		

rabbit ATR	Cell Signaling Technology	2790; RRID:AB_2227860
rabbit BRCA1	gift from S. Namekawa	N/A
rat CBX1	gift from P. B. Singh	N/A
human centromere (CREST serum)	gift from W. Earnshaw	N/A
rabbit cleaved-PARP	Abcam	ab32064; RRID:AB_777102
rabbti GFP	Cell Signaling Technology	2956; RRID:AB_1196615
rabbit H3K9me3 (for ChIP)	Active Motif	39161; RRID:AB_2532132
rabbit H3K9me3 (for IF)	Millipore	07-442; RRID:AB_310620
rabbit hnRNP K	Abcam	ab52600; RRID:AB_880478
guinea pig HORMAD2	gift from A. Tóth	N/A
sheep MDC1	Serotec	AHP799; RRID:AB_323725
mouse poly-ubiquitylation (clone E6C5)	Millipore	05-678; RRID:AB_11214408
rabbit RAD51	Millipore	PC130; RRID:AB_2238184
rabbit RPA2	Abcam	ab10359; RRID:AB_297095
rabbit SETDB1	Proteintech	11231-1-AP; RRID:AB_2186069
guinea pig SYCP3	in house	N/A
rabbit TOPBP1	Abcam	ab105109; RRID:AB_11129928
mouse TRIM28	Abcam	ab22553; RRID:AB_447151
rabbit USP7	Bethyl	A300-033A; RRID:AB_203276
mouse α-Tubulin	Sigma	T9026; RRID:AB_477593
mouse γH2AFX	Millipore	05-636; RRID:AB_309864
VeriBlot for IP Detection Reagent (HRP)	Abcam	ab131366
Anti-mouse IgG VeriBlot for IP secondary antibody (HRP)	Abcam	ab131368

**Deposited Data**		

RNA-seq and ChIP-seq data	this study	GEO: GSE107671

**Experimental Models: Organisms/Strains**		

*Hormad2* KO	Dr. A. Tóth	Wojtasz et al. (2012)
*Brca1*^*Δ11*^	NCIMR	*Brca1*^*tm2.1Cxd*^
*H2afx* KO	Dr. A. Nussenzweig	Celeste et al. (2002)
*Dmc1* KO	Jackson laboratory	*Dmc1*^*tm1Jcs*^
*Atr*^*flox*^	Jackson laboratory	*Atr*^*tm2Bal*^
*Setdb1*^*flox*^	Dr. Y. Shinkai	Matsui et al. (2010)
*Gt(ROSA)26Sor*^*tm1(EYFP)Cos*^	Jackson laboratory	*Gt(ROSA)26Sor*^*tm1(EYFP)Cos*^
*Ngn3-Cre*	Jackson laboratory	Tg(Neurog3-cre)C1Able
*Stra8-Cre*	Jackson laboratory	Tg(Stra8-icre)1Reb

**Software and Algorithms**		

Fiji	http://fiji.sc/	N/A
R	https://www.r-project.org/	v3.3.2
Prism	GraphPad	v7.0d
Kallisto	https://pachterlab.github.io/kallisto/	v0.44.0
Sleuth	https://pachterlab.github.io/sleuth/	v0.30.0
BiomaRt	https://bioconductor.org/packages/biomaRt/	v2.36.1
DAVID Bioinformatics Resources	https://david.ncifcrf.gov	v6.8
ChromHeatMap	https://bioconductor.org/packages/ChromHeatMap/	v1.34.0
AnnotationDbi	https://bioconductor.org/packages/AnnotationDbi/	v1.42.1
org.Mm.eg.db	https://bioconductor.org/packages/org.Mm.eg.db/	v3.6.0
pairwiseCI	https://cran.r-project.org/web/packages/pairwiseCI/index.html	v0.1.26
Hisat2	https://ccb.jhu.edu/software/hisat2/index.shtml	v2.1.0
deepTools	https://deeptools.readthedocs.io/en/develop/	v3.1.1
RepEnrich	https://github.com/nskvir/RepEnrich	v1.2
DESeq2	https://bioconductor.org/packages/DESeq2/	v1.20.0
MaxQuant	http://www.coxdocs.org/doku.php?id=:maxquant:start	v1.6.0.13
Perseus	http://www.coxdocs.org/doku.php?id=perseus:start	v1.4.0.2

**Other**		

Published RNA-seq datasets reanalysed in this study	GEO	GSE35005, GSE60377, GSE29413

### Contact for Reagent and Resource Sharing

Further information and requests for reagents should be directed to and will be fulfilled by the Lead Contact, James M.A. Turner (james.turner@crick.ac.uk).

### Experimental Model and Subject Details

#### Mice

All animals were maintained with appropriate care according to the United Kingdom Animal Scientific Procedures Act 1986 and the ethics guidelines of the Francis Crick Institute. Mice were housed in individually ventilated cages and had free access to water and food. All studies were approved by local ethical review and UK Home Office. Male mice at age indicated in figures or legends were used for analyses. C57BL/6J strain was used as wildtype. *Hormad2* KO mice (Wojtasz et al., 2012), *Brca1*^*Δ11*^ mice (Xu et al., 1999), *H2afx* KO mice (Celeste et al., 2002), and *Dmc1* KO mice (Pittman et al., 1998) were generated on the C57BL/6J background. *Atr* cKO mice (Widger et al., 2018) were generated on the C57BL/6J background by mating *Atr*^*flox/flox*^ females (Brown and Baltimore, 2003) with *Atr*^*+/-*^ (Brown and Baltimore, 2003); *Ngn3-Cre* (Schonhoff et al., 2004) males. *Setdb1* control and cKO mice were generated on the C57BL/6J background by mating *Setdb1*^*flox/flox*^; *Gt(ROSA)26Sor*^*tm1(EYFP)Cos/tm1(EYFP)Cos*^ (Matsui et al., 2010, Srinivas et al., 2001) females with *Setdb1*^*+/-*^ (Matsui et al., 2010) males carrying either *Ngn3-Cre* or *Stra8-Cre* (Sadate-Ngatchou et al., 2008) transgene.

### Method Details

#### Immunofluorescence Staining of Nuclear Spreads

Glass slides (ThermoFisher, AA00008032E00MNT10) were cleaned by boiling in water for ten minutes and dried before use. Testes were dissected in Roswell Park Memorial Institute (RPMI) medium and 100 μl of cell suspension were placed on slides. Slides were applied with 50 μl of 0.05% Triton X-100 in water and incubated at room temperature for 10 minutes. Cells were fixed in 2% paraformaldehyde (PFA), 0.02% sodium dodecyl sulfate (SDS) in phosphate-buffered saline (PBS) at room temperature for one hour, washed in water, and air-dried. Slides were blocked in blocking solution [0.15% bovine serum albumin (BSA) and 0.1% Tween 20 in PBS] at room temperature for one hour and incubated with primary antibodies (see list in Key Resources Table) in humidified chamber at 37°C overnight. For TRIM28 staining, slides were incubated at 4°C. Dilution of primary antibodies were 1:10 (MDC1, TRIM28), 1:50 (ATR, RAD51, RPA2), 1:100 (CREST, H3K9me3, hnRNP K, HORMAD2, SETDB1, SYCP3, TOPBP1), 1:200 (CBX1, poly-Ub, USP7), 1:250 (γH2AX), 1:500 (BRCA1). Secondary antibodies (1:250-500, AlexaFluor 488, 568, or 647, ThermoFisher) were applied in blocking buffer at 37°C for one hour. After wash in PBS at room temperature, specimens were mounted in Vectashield with 4',6-Diamidino-2-phenylindole (DAPI; Vector Laboratories, H-1200).

#### Plot Profiles of H3K9me3 and γH2AX Staining

Captured images of nuclear spreads immunostained for SYCP3, γH2AX, H3K9me3 were analysed using Fiji (Rueden et al., 2017, Schindelin et al., 2012). Signal intensity at the origin was assigned a value of 1, and relative intensity was calculated.

#### Measurement of Normalized Distance between X- and Y-Centromeres

Captured images of nuclear spreads immunostained for SYCP3, HORMAD2 and centromere were analysed using Fiji (Rueden et al., 2017, Schindelin et al., 2012). Normalised distance was calculated by dividing measured distance between X- and Y-centromeres by largest diameter of nucleus.

#### RNA Fluorescence In Situ Hybridization (RNA-FISH) Followed by Immunofluorescence Staining

Bacterial artificial chromosome probes for *Scml2* (RP24-204O18, Children's Hospital Oakland Research Institute) and *Zfy2* (CITB-288D7, Research Genetics) were labelled using Abbott Nick Translation Kit. Testes were dissected in RPMI medium and 100 μl of cell suspension was placed on cleaned glass slides (kept cold hereafter). Cells were permeabilised in 0.5% Triton X-100, 2 mM Vanadyl Ribonucleoside in PBS for 10 minutes, fixed in 4% PFA in PBS for 10 minutes and washed in PBS. After dehydration in an ethanol series (2x 70, 80, 95, 100%), air-dried specimens were hybridised with the denatured probe mixed with 3 μg of mouse Cot-1 DNA (ThermoFisher, 18440016) in hybridisation buffer (50% formamide, 25% dextran sulphate, 5 mg/ml bovine serum albumin, 1mM Vanadyl Ribonucleoside in 2x SSC) at 37°C overnight. Slides were washed in 50% Formamide in 1x SSC at 45°C and in 2x SSC at 45°C, and then blocked in 1% BSA, 0.1% Tween in 4x SSC at 37°C for 30 minutes. Slides were incubated with HORMAD2 antibody at 37°C for 30 minutes. Secondary antibody (1:250, AlexaFluor 647, ThermoFisher) was applied in PBS at 37°C for 30 minutes. After wash in 0.1% Tween in 4x SSC at room temperature, specimens were mounted in Vectashield with DAPI.

#### DNA-FISH

DNA-FISH of nuclear spread specimens was performed as described in detail previously (Hirota et al., 2017). Briefly, specimens were washed in 2x SSC and denatured in 70% formamide in 2x SSC. After dehydration in an ethanol series, air-dried specimens were hybridised with the denatured probe in hybridisation buffer at 37°C for overnight. After washing in 2x SSC, 0.1x SSC, and 4xSSC, 0.1 % Tween20, specimens were mounted in Vectashield with DAPI. Bacterial artificial chromosome [RP24-502P5 (*Sly*)] or chromosome painting probes from MetaSystems (XMP 1 Green and XMP 19 Orange) were used as probes.

#### Immunofluorescence Staining of Testis Sections

Isolated testes were fixed in 4% PFA at 4°C overnight, washed in 70% ethanol, embedded in paraffin, and sectioned. Sections attached on glass slides were warmed at 60°C for 10 minutes and washed in Histo-Clear (National Diagnostics, HS-200) at room temperature for five minutes twice. Specimens were rehydrated in an ethanol series (2x 100, 2x 95, 75%) and then in water. Antigen retrieval was performed by boiling the slides in 0.01 M sodium citrate in water for 10 minutes and letting the solution cool for 20 minutes. Slides were blocked in blocking solution (5% normal goat serum and 0.1% Triton X-100 in PBS) at room temperature for one hour and incubated with primary antibodies (see list in Key Resources Table) in humidified chamber at 37°C for two hours. Dilution of primary antibodies were 1:250 (cleaved-PARP, γH2AX). Secondary antibodies (1:500, AlexaFluor 488, 568, ThermoFisher) were applied in blocking buffer at room temperature for one hour. After wash in PBS at room temperature, specimens were mounted in Vectashield with DAPI.

For EYFP staining, testes were fixed in 4% PFA at 4°C for two hours, washed in 0.2% Tween 20 in PBS, immersed successively in 10% and 30% sucrose in PBS, embedded in OCT compound (VWR), frozen, and sectioned at -20°C. Dried specimens were washed three times in PBS, blocked in blocking solution at room temperature for 30 minutes and incubated with GFP antibody (1:200) at 4°C overnight. Secondary antibody (1:500, AlexaFluor 488 anti-rabbit IgG, ThermoFisher) was applied in blocking buffer at room temperature for one hour. After wash in PBS, specimens were mounted in Vectashield with DAPI.

#### Histology

Isolated testes and epididymides were fixed in Bouin’s solution overnight, washed in 70% ethanol, embedded in paraffin, sectioned, and stained with Periodic Acid-Schiff staining (for testes) or hematoxylin and eosin (for epididymides).

#### Microscopy

Images of nuclear spreads, RNA-FISH, and DNA-FISH were captured using Deltavision Microscopy System (100x/1.35NA Olympus UPlanApo objective; GE Healthcare). Images of immunostained testis sections were captured using Leica SP5 confocal microscopy (63x/1.40NA objective). Histology images were captured using Olympus BH2 microscope with 20x/0.46NA (for epididymis) or 40x/0.70NA (for testis) Olympus SPlan objectives.

#### Isolation of Pachytene Spermatocytes

Early-to-mid pachytene cells were purified using fluorescence activated cell sorting following the published method (Bastos et al., 2005) with a modification from testes of wildtype C57BL/6J mice at postnatal day 15 (P15), when germ cells reach early-mid pachynema in the first wave of spermatogenesis. Briefly, seminiferous tubules were dissociated by two-step collagenase digestions and treated with Trypsin buffer (0.125% Trypsin, 500 μg/ml collagenase, 3.29 μg/ml DNase I in the incubation buffer described in Bastos et al., 2005) at 32°C for 20 minutes. Cell suspension was filtered (40 μm), stained with Hoechst 33342 (5 μg/ml) and propidium iodide (2 μg/ml), and used for sorting. As spermatocytes are arrested at midpachynema in *Setdb1* cKOs, *Setdb1* cKO mice at P15 or later were used for isolating early-to-mid pachytene cells. Immunofluorescence staining of nuclear spreads using test-sorted testicular cells from P15 wildtype mice confirmed purity of pachytene cells (82% and 76%, 50 cells analysed for each). 500 cells for each genotype were used for RNA isolation. 3268 cells of wildtype and 5203 cells of *Setdb1* cKO were used for chromatin immunoprecipitation.

#### RNA Sequencing (RNA-Seq)

Total RNA was extracted from 500 pachytene cells using RNAqueous-Micro Total RNA Isolation Kit (ThermoFisher, AM1931) and eluted in 10 μl of elution solution. cDNA was amplified from 1 μl of total RNA by using Smart-seq2 protocol (Picelli et al., 2013, Picelli et al., 2014) (15 amplification cycles). 10 ng of cDNA was sheared using Covaris E220. Libraries of ∼300 bp were generated using Ovation Ultralow DR kit (NuGEN, 0330) with 14 PCR cycles. Sequencing was performed on the Illumina HiSeq 4000 system (single read, 75 bp).

#### Chromatin Immunoprecipitation Sequencing (ChIP-Seq)

Ultra-low-input native ChIP-seq libraries were prepared by following a published protocol (Brind’Amour et al., 2015) using sorted wildtype (3268 cells) and *Setdb1* cKO (5203 cells) early-to-mid pachytene cells. Briefly, sorted cells were treated with micrococcal nuclease at 21°C for 7.5 minutes and used for H3K9me3 ChIP. 5% of the samples after micrococcal nuclease digestion were taken as input. 6.25 μl of 1:100 diluted H3K9me3 antibody (Active Motif, 39161) per reaction was used for ChIP. DNA from pulled-down chromatin was eluted, end-repaired, phosphorylated, and A-tailed. NEBNext Adapter for Illumina was used as adapters and adapter-ligated DNAs were amplified by 13 PCR cycles using NEBNext Index primers (New England Biolabs, included in E7335 and E7500). The libraries were size-selected (200-500 bp) using E-Gel (ThermoFisher). Sequencing was performed on the Illumina HiSeq 2500 system (single read, 75 bp).

#### Immunoprecipitation (IP)

Four testes of wildtype C57BL/6J mice at P15 were homogenised in 1 ml of IP buffer [20 mM Tris (pH 7.5), 150 mM sodium chloride, 1% Triton X-100, 25 U/ml Pierce Universal Nuclease (ThermoFisher, 88700), supplemented with protease inhibitor cocktail (Roche, 04693159001) and phosphatase inhibitor cocktail (Roche, 04906845001)] and rotated at room temperature for 30 minutes. Lysates were cleared by centrifugation (15000 g, 4°C for 10 minutes) twice and incubated with 20 μl of 1:1 mixture of protein A and protein G Dynabeads (ThermoFisher, 10002D and 10004D; pre-washed in IP buffer) at 4°C for 30 minutes. After removing beads, 400 μl of precleared lysates were mixed with 3 μg of antibodies and rotated at 4°C for 1.5 hours. Lysates were then mixed with 50 μl of 1:1 mixture of protein A and protein G Dynabeads (pre-washed in IP buffer) and rotated at 4°C overnight. Beads were washed five times in IP buffer without nuclease at 4°C and boiled in 1x SDS-PAGE sample buffer [80 mM Tris (pH6.8), 2% SDS, 10% Glycerol, 0.0006% Bromophenol blue, 5% 2-Mercaptoethanol] for 10 minutes.

#### Western Blot

For the data used in Figures 2G and S2B, testes were homogenised in lysis buffer [1mM Tris (pH 8.0), 150 mM sodium chloride, 1mM ethylene glycol-bis(2-aminoethylether)-N,N,N',N'-tetraacetic acid (EGTA), 1 mM magnesium chloride, 0.1% NP40, 1mM dithiothreitol (DTT), 1mM phenylmethanesulfonyl fluoride (PMSF), supplemented with protease inhibitor cocktail (Roche)], incubated on ice for 15 minutes, and the resulting lysates were cleared by centrifugation (6000 g, 4°C for 10 minutes).

Protein lysates were used for SDS-PAGE (Laemmli, 1970) and blotted onto polyvinylidene difluoride membranes (Millipore, IPVH00005) or nitrocellulose membranes (LI-COR, 926-31092; used only for γH2AX detection). Membranes were blocked in blocking buffer (5% skimmed milk in TBST) at room temperature for one hour and incubated with primary antibodies (see list in Key Resources Table) in blocking buffer at 4°C overnight. Dilution of primary antibodies were 1:1000 (SETDB1, γH2AX, TRIM28) and 1:5000 (α-Tubulin). Secondary antibody (VeriBlot for IP Detection Reagent or Anti-mouse IgG VeriBlot for IP secondary antibody, 1:3000, Abcam) was applied in blocking buffer at room temperature for 45 minutes. After wash in TBST, signals were detected using Clarity Western ECL Substrate (Bio-Rad, 1705060).

#### Trypsin Digestion

The immunoprecipitated proteins were electrophoresed for approximately 1 cm by SDS-PAGE. Whole lanes were then excised and proteins were subjected to in-gel trypsin digestion using a Perkin Elmer Janus liquid handling system. Briefly, the excised gel pieces were de-stained in 50% acetonitrile, 50 mM ammonium bicarbonate. Cysteines were reduced in 10 mM DTT, and alkylated in 55 mM iodoacetamide. After alkylation, the proteins were digested with 6 ng/μl trypsin at 37°C overnight. The resulting peptides were extracted in 2% formic acid, 1% acetonitrile.

#### Mass Spectrometry

The peptides were loaded on 50-cm Easy Spray column (75 μm inner diameter, 2 μm particle size, ThermoFisher), equipped with an integrated electrospray emitter. Reverse phase chromatography was performed using the RSLC nano U3000 (ThermoFisher) with a binary buffer system at a flow rate of 250 nl/minute. Solvent A was 0.1% formic acid, 5% DMSO, and solvent B was 80% acetonitrile, 0.1% formic acid, 5% DMSO. The in-gel digested samples were run on a linear gradient of solvent B (2- 35%) for 98 minutes. Total run time was 120 minutes including column conditioning. Each sample was analysed in triplicate. The nanoLC was coupled to an LTQ Orbitrap Velos Pro (ThermoFisher) operated in data-dependent mode acquiring one MS1 scan followed by 10 CID scans acquired in centroid mode. The CID normalized collision energy was set at 35 with 10 milliseconds activation time and a maximum ion injection time for MS2 scans at 50 milliseconds. The dynamic exclusion was set at 20 seconds and singly charged peptides and peptides with unassigned charge states were excluded from fragmentation.

### Quantification and Statistical Analysis

#### Statistics and Reproducibility

R v3.3.2 and GraphPad Prism 7 were used for statistical tests. Lines on dot plots show mean values. Box plots are defined in figure legends. Two-tailed p-values were calculated using unpaired t-test (Figures 2F, 2K, 4B, and S2A), Mann-Whitney test (Figures 4A, 6D, and S3), Welch's t-test (Figures 5B, S5B, S5C, and S7B), or Fisher's exact test (Figures S4A and S4D). P-values <0.05 were regarded as significant. The experimental findings were reproduced successfully at least twice. Due to limited cell number, ChIP-seq experiment was performed on single wildtype and *Setdb1* cKO samples though four technical replicates were performed for each sample.

#### Analysis of RNA-Seq Data

RNA-seq data were processed using an R script and transcript quantification was performed using the kallisto R package (Bray et al., 2016). Kallisto was run in single-end read mode using the mouse transcriptome index (GRCm38), with the estimated average fragment length set to 200, the average standard deviation of fragment length set to 20, and the number of bootstraps for estimating confidence intervals set to 150. Paired-end mode was selected for the PGC *Setdb1* cKO dataset (Liu et al., 2014). Normalisation was performed using kallisto to generate transcript-level TPM values which were later aggregated to gene-level TPM values using sleuth (Pimentel et al., 2017). Protein-coding genes having an average TPM >0.1 in at least one condition were used in subsequent scatterplot and boxplot analyses. Differential expression analysis was performed using sleuth and genes with q-value <0.2 calculated by Wald test were considered as differentially expressed. All chromosome and gene biotype annotations were extracted from Ensembl using the R package BiomaRt (Durinck et al., 2009). Gene ontology analyses were performed using DAVID Bioinformatics Resources (https://david.ncifcrf.gov) (Huang et al., 2009a, Huang et al., 2009b).

#### Chromosome Heatmap

The R package ChromHeatMap (http://bioconductor.org/packages/ChromHeatMap/) was used to plot the log_2_ expression ratio of *Setdb1* cKO relative to wildtype TPM values as a heatmap separately for both X and Y chromosomes. The location of each gene along the heatmap was precisely mapped to the genomic locus using the AnnotationDbi and org.Mm.eg.db packages (http://bioconductor.org/packages/AnnotationDbi/; https://www.bioconductor.org/packages/org.Mm.eg.db/).

#### X-Autosome Expression Ratio

X:autosome expression ratios with 95% confidence intervals were calculated using the pairwiseCI package (https://cran.r-project.org/web/packages/pairwiseCI/index.html) in R using ’median.ratio’ setting with 1,000,000 bootstrap replications. Genes that were expressed < 1 TPM were excluded from downstream calculations.

#### Analysis of ChIP-Seq Data

Sequences were aligned using Hisat2 v2.1.0 (Kim et al., 2015) to the mouse genome index (GRCm38). Coverage from each bam file was computed using the deepTools (Ramírez et al., 2014) and compared in 100 bp genomic bins after normalisation for sequencing depth with setting ‘reciprocal ratio’. Gene regions were scaled to the same size, and 3 kb upstream and downstream sequences were included for computing scores for each region in comparisons. This score matrix was used to generate profile plots for each set of genomic regions.

#### ERV Analysis Using RNA-Seq and ChIP-Seq Data

Sequences were aligned to the genome using Hisat2 (v2.1.0), and unique and multi-mapped reads were extracted separately. Spliced alignments were permitted on RNA-seq libraries, but disallowed for ChIP-seq libraries. Annotation for *M. musculus* repetitive LINE and LTR elements were obtained from UCSC genome Table Browser (mm10). The RepEnrich package (Criscione et al., 2014) was subsequently used to obtain fraction counts for each repetitive element, and used in further downstream analyses. Estimated read counts from the RNA-seq data were used to perform differential expression analysis between *Setdb1* cKO and wildtype samples using DESeq2 (Love et al., 2014). A negative binomial distribution was applied to the count data using the parametric mode, and significance test was performed using the Wald test. Genes having a p-value < 0.01 were labelled significant on subsequent scatter plots comparing counts per million (CPM) between *Setdb1* cKO and wildtype samples.

#### Processing and Analysis of Mass Spectrometry Data

Raw data files were analysed using MaxQuant software (version 1.6.0.13) (Cox and Mann, 2008). Parent ion and tandem mass spectra were searched against UniprotKB *Mus musculus* (July 2017) database. For the search the enzyme specificity was set to trypsin with maximum of two missed cleavages. The precursor mass tolerance was set to 20 ppm for the first search (used for mass re-calibration) and to 6 ppm for the main search. Product mass tolerance was set to 0.5 Da. Carbamidomethylation of cysteines was specified as fixed modification, oxidized methionines and N-terminal protein acetylation were searched as variable modifications. The datasets were filtered on posterior error probability to achieve 1% false discovery rate on protein level. For label free quantification the LFQ option within MaxQuant software was selected. The proteingroups.txt MaxQuant output table was imported into Perseus v1.4.0.2 software (Tyanova et al., 2016) for further downstream processing and statistical analysis. LFQ intensities were log_2_ transformed and missing values were imputed from a noise distribution generated using default Perseus settings. LFQ values were then used to determine proteins that were significantly enriched compared to the control (IgG) conditions. The test settings were Welch's t-test, S0=1.0 and a permutation-based FDR controlled at 0.01.

### Data and Software Availability

The sequencing datasets generated during this study are available in the Gene Expression Omnibus (GEO) under the accession code GEO: GSE107671 (https://www.ncbi.nlm.nih.gov/geo/query/acc.cgi?acc=GSE107671). GEO accession codes of the published RNA-seq datasets re-analysed during this study are GEO: GSE35005 (Figures 2A and S5A) (Gan et al., 2013), GEO: GSE29413 (Figure S5B) (Karimi et al., 2011), and GEO: GSE60377 (Figure S5C) (Liu et al., 2014).

## Supplemental Information

Document S1. Figures S1–S7

Table S1. List of Differentially Expressed Genes upon *Setdb1* Deletion, Related to Figure 5

Table S2. List of Gene Ontology Terms Enriched in Differentially Expressed Genes upon *Setdb1* Deletion, Related to Figure 5

Table S3. List of Differentially Expressed ERVs upon *Setdb1* Deletion, Related to Figure S7

Table S4. List of Proteins Identified in IP-MS Experiments, Related to Figure 6

Document S2. Article plus Supplemental Information
