# Supplementary material for: SETDB1 Links the Meiotic DNA Damage Response to Sex Chromosome Silencing in Mice
Source: Dev Cell. 2018 Dec 3;47(5):645–659.e6. doi: 10.1016/j.devcel.2018.10.004 (PMC6286383; doi:10.1016/j.devcel.2018.10.004)
Supplement: Document S1. Figures S1–S7 [file mmc1.pdf]

Developmental Cell, Volume *47*

Supplemental Information

SETDB1 Links the Meiotic DNA Damage Response to Sex Chromosome Silencing in Mice

Takayuki Hirota, Paul Blakeley, Mahesh N. Sangrithi, Shantha K. Mahadevaiah, Vesela Encheva, Ambrosius P. Snijders, Elias ElInati, Obah A. Ojarikre, Dirk G. de Rooij, Kathy K. Niakan, and James M.A. Turner

Supplemental Figures:

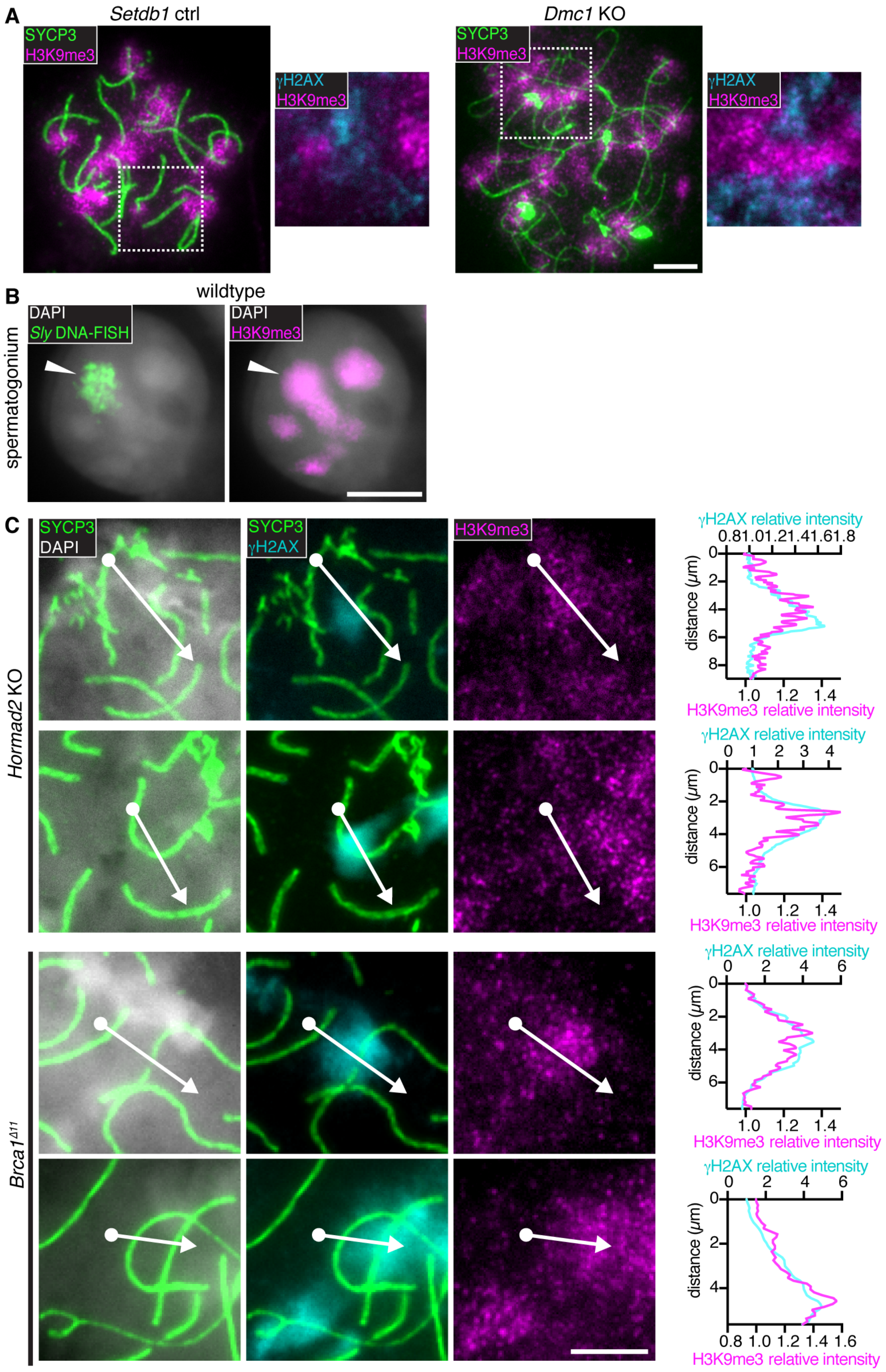

Figure S1. Analyses of H3K9me3 localisation in male germ cells (related to Figure 1)

(**A**) Early pachytene spermatocytes from *Setdb1* control (n = 30 cells) and *Dmc1* KO (n = 30 cells) immunostained for SYCP3 (green), H3K9me3 (magenta), and γH2AX (cyan). Dashed rectangles highlight regions magnified to show persistent γH2AX in each genotype.

(**B**) *Sly* DNA-FISH (green) of spermatogonia from wildtype (n = 30 cells) immunostained for H3K9me3 (magenta). Arrowheads indicate position of Y chromosome.

(**C**, related to Figure 1C and 1D) Other examples of ectopic γH2AX-positive region overlapping with H3K9me3 in *Hormad2* KO and *Brca1*$^{\Delta 11}$ pachytene cells. SYCP3: green. γH2AX: cyan. H3K9me3: magenta. Arrows show lines used for plot profile analysis.

8-week old mice were used for analyses. Scale bars: 5 μm.

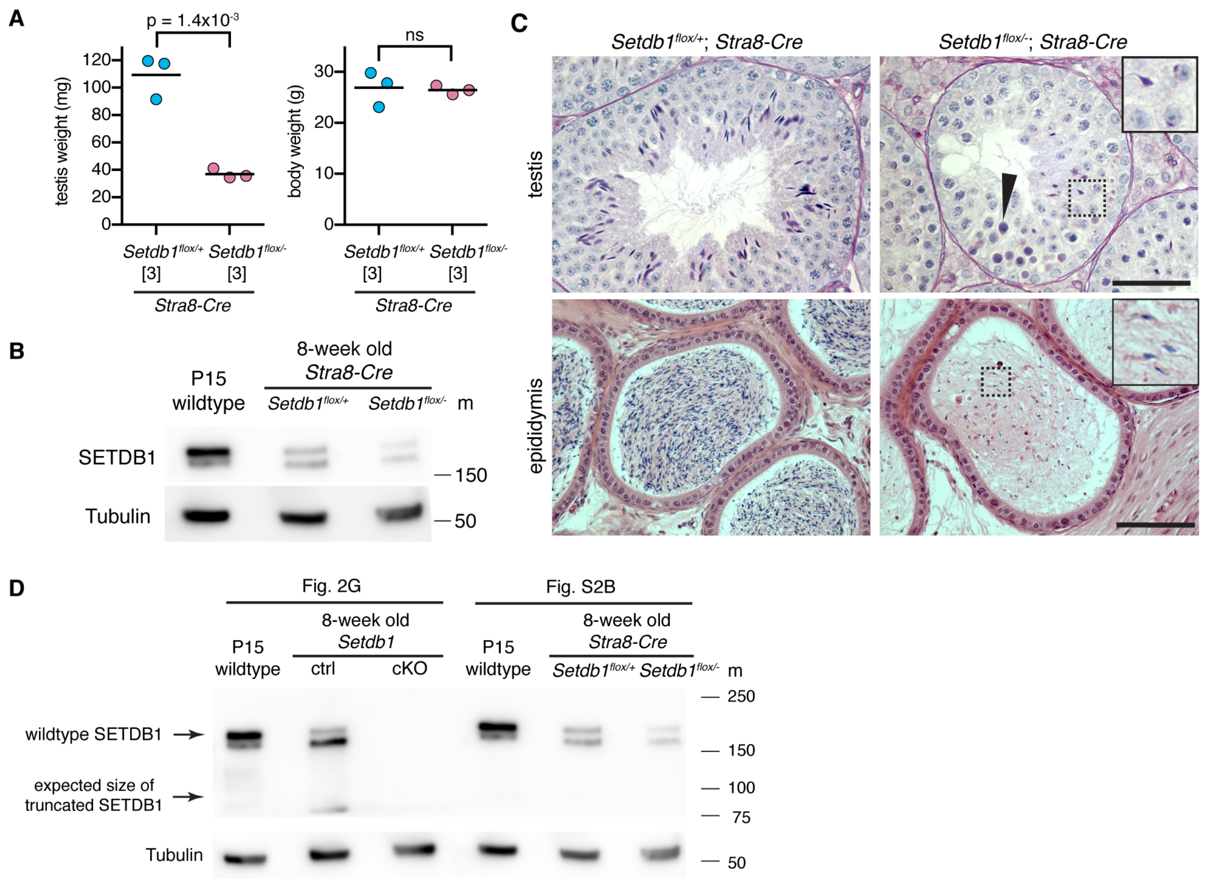

Figure S2. *Stra8-Cre* is less efficient than *Ngn3-Cre* at conferring *Setdb1* deletion (related to Figure 2)

(**A**) Testis and body weights in *Setdb1*$^{flox/+}$; *Stra8-Cre* and *Setdb1*$^{flox/-}$; *Stra8-Cre* mice. Number of mice analysed in brackets. ns: not significant. p-value calculated using unpaired t-test.

(**B**) Testis SETDB1 western blot of *Setdb1*$^{flox/+}$; *Stra8-Cre* and *Setdb1*$^{flox/-}$; *Stra8-Cre*. Tubulin was used as a loading control. 50 μg of protein per lane was loaded. m: size marker. Expected size: 180 kDa (SETDB1), 50 kDa (Tubulin). SETDB1 antibody used recognises two SETDB1 bands.

(**C**) Histology of *Setdb1*$^{flox/+}$; *Stra8-Cre* and *Setdb1*$^{flox/-}$; *Stra8-Cre* testes (Periodic Acid-Schiff staining) and epididymides (hematoxylin and eosin staining). Dashed rectangles highlight

haploid round/elongated spermatids (testis) and sperm (epididymis), which are magnified in insets. Scale bars: 50 μm (testis), 100 μm (epididymis).

(**D**) A wider view of the testis SETDB1 western blot images shown in Fig. 2G and S2B. m: size marker. Expected size: 180 kDa (SETDB1), 88.7 kDa (truncated SETDB1), 50 kDa (Tubulin). SETDB1 antibody used is expected to recognise the SETDB1 N-terminal region, which is also present in the truncated SETDB1 expressed after Cre recombination. This shorter SETDB1 was not detected in the *Setdb1* control and cKO.

8-week old mice were used for analyses except for P15 sample in **B** and **D**.

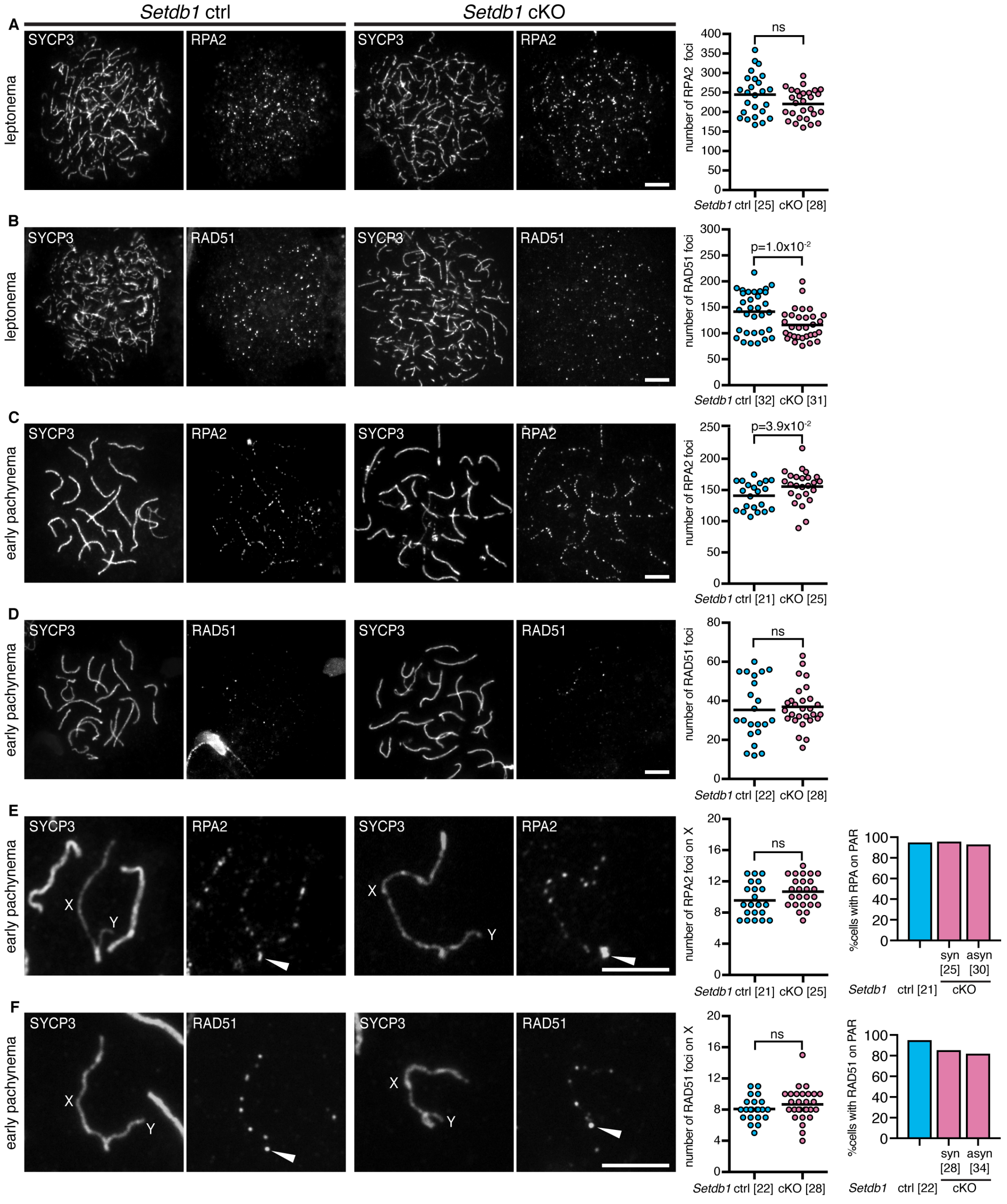

Figure S3. Minor meiotic recombination defects in *Setdb1* cKOs (related to Figure 3)

Immunostaining and quantification of RPA2 (**A, C, E**) or RAD51 (**B, D, F**) foci in *Setdb1* control and cKO leptotene (**A, B**) and early pachytene (**C-F**) spermatocytes. Magnified images of XY pairs are shown in **E** and **F**. Bar charts show percentage of cells positive for RPA2 (**E**) and RAD51 (**F**) on PAR. syn: XY synapsed. asyn: XY asynapsed. Number of cells analysed in

brackets. ns: not significant. p-value calculated using Mann-Whitney test. 8-week old mice were used for analyses. Scale bars: 5 μm.

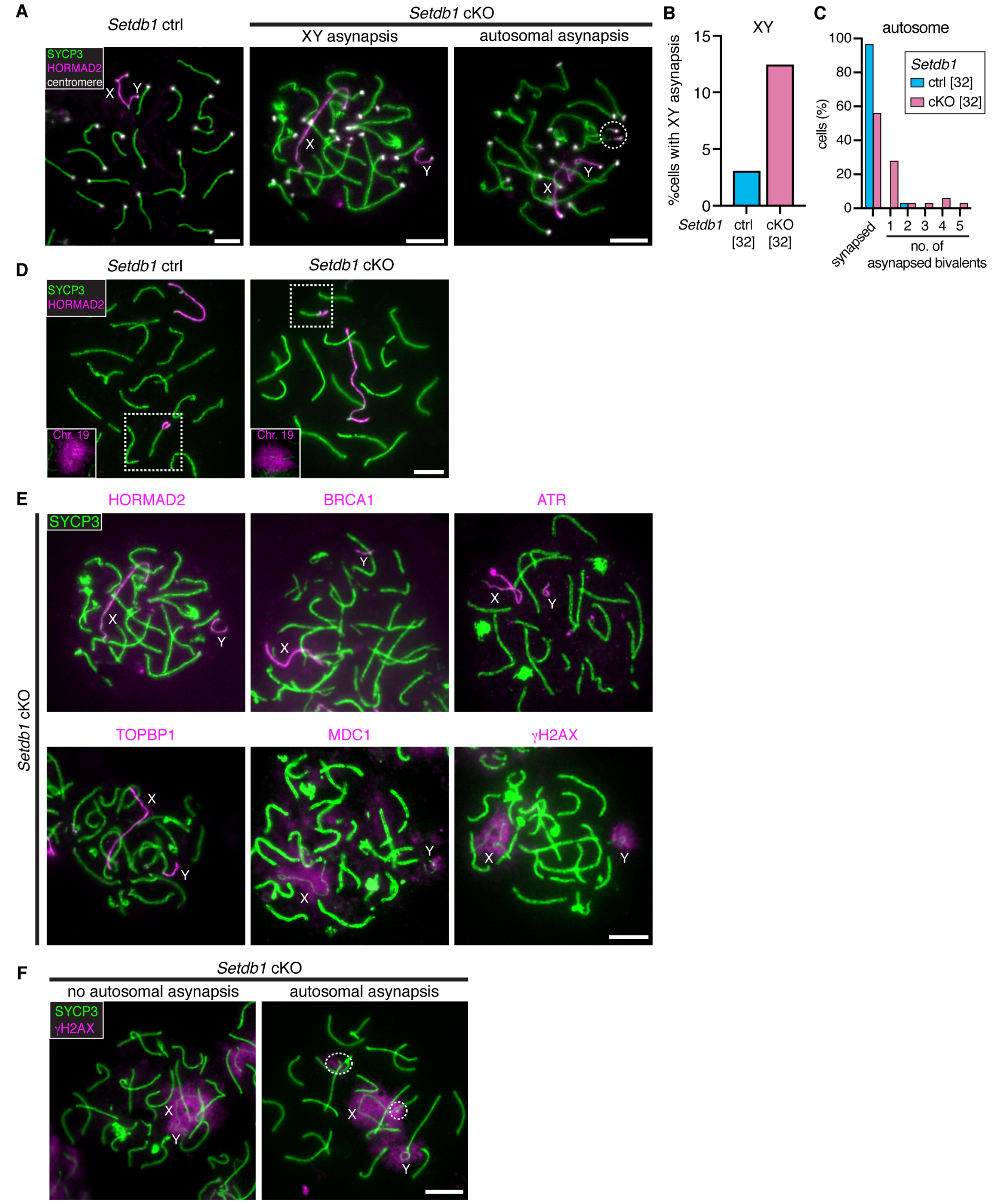

Figure S4. Mild synapsis defects at early pachynema in *Setdb1* cKOs (related to Figure 3)

(**A**) *Setdb1* control and cKO spermatocytes immunostained for SYCP3 (green), HORMAD2 (magenta), and centromeres (white). Dashed circle: autosomal bivalent with asynapsis at centromeric end.

(**B**) Percentage of cells with XY asynapsis. Number of cells analysed in brackets.

(**C**) Percentage of cells with autosomal pairs with asynapsis at centromeric end. Number of cells analysed in brackets. Asynapsis at centromeric end was more common in *Setdb1* cKOs than controls ($p = 1.5 \times 10^{-4}$, Mann-Whitney test).

(**D**) *Setdb1* control and cKO spermatocytes immunostained for SYCP3 (green) and HORMAD2 (magenta), followed by DNA-FISH using chromosome paint. Dashed rectangles highlight asynapsed chromosome 19, of which DNA-FISH images are shown in insets (magenta). Cells with autosomal asynapsis were used for quantitation. In both *Setdb1* controls and cKOs, chromosome 19 (*Setdb1* control: 11 out of 21 cells; *Setdb1* cKO: 14 out of 35 cells) showed significantly more asynapsis than chromosome 1 (*Setdb1* control: 1 out of 21, $p = 7.2 \times 10^{-4}$, Fisher's exact test; *Setdb1* cKO: 3 out of 35, $p = 3.0 \times 10^{-3}$, Fisher's exact test).

(**E**, related to Figure 3A) Early pachytene *Setdb1* control and cKO asynapsed XY immunostained for SYCP3 (green) and silencing factors (magenta; $n \geq 30$ cells for each factor).

8-week old mice were used for analyses. Scale bars: 5 μm.

(**F**) *Setdb1* cKO spermatocytes immunostained for SYCP3 (green) and γH2AX (magenta). Left: no autosomal asynapsis (n = 20 cells). Right: autosomal asynapsis at centromeric end (dashed circle, n = 20 cells).

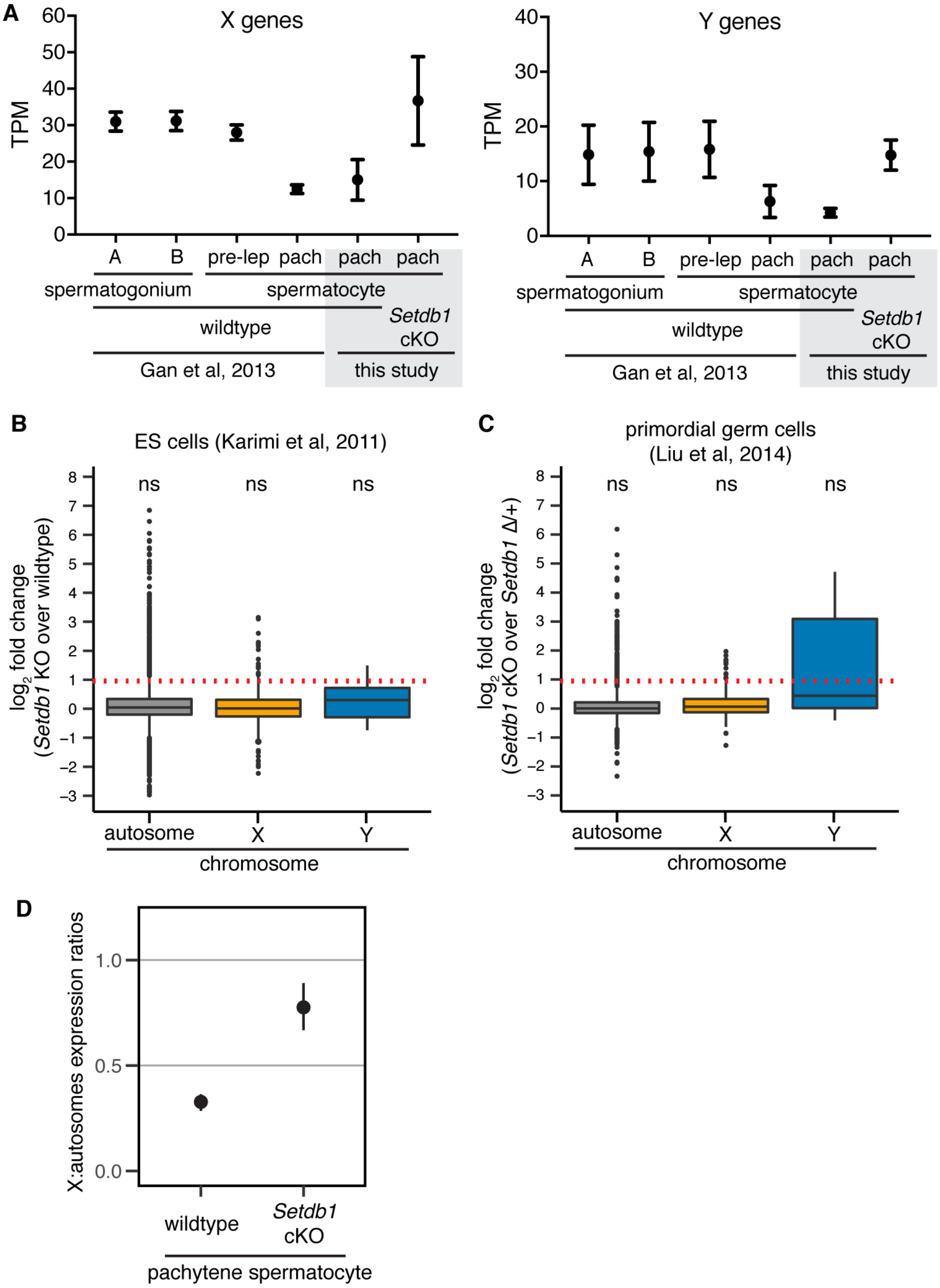

Figure S5. Comparison of RNA-seq datasets generated in this study and published studies (related to Figure 5)

(**A**) XY gene expression levels in male germ cells by RNA-seq (A: type A, B: type B, pre-lep: pre-leptonema, pach: pachynema, TPM: transcripts per million). Charts show mean TPM ± standard error. Genes having an average TPM > 0.1 in at least one condition at pachynema were used for visualisation.

(**B-C**) Box plots represent gene expression log_2 fold change of *Setdb1* KO ES cells relative to wildtype (**B**) or of *Setdb1* cKO primordial germ cells relative to control (*Setdb1* Δ/+) (**C**). Box: 25th/75th percentiles. Line on box: median. Whisker: 1.5 times the interquartile range from the 25th/75th percentiles. Red dashed line: 2-fold change. ns: not significant. p-value calculated using Welch's t-test.

(**D**) Median gene expression level ratio of X genes relative to autosomal genes in wildtype and *Setdb1* cKO pachytene cells. 231 X-linked and 8198 autosomal genes (TPM > 1) were used for calculation. Error bars show 95% confidence intervals.

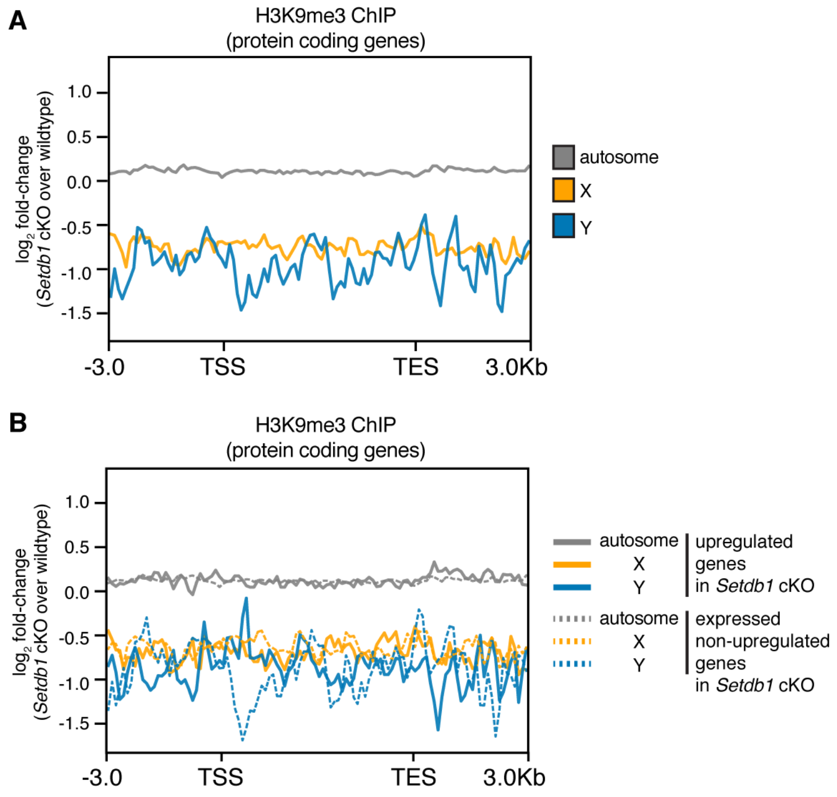

Figure S6. H3K9me3 occupancy changes in protein coding genes in *Setdb1* cKOs (related to Figure 5)

(**A-B**) H3K9me3 occupancy $\log_2$ fold-change of *Setdb1* cKO relative to wildtype in protein coding genes. All protein coding genes are shown in **A**. Protein coding genes upregulated in *Setdb1* cKO and expressed non-upregulated genes (dashed lines; average TPM >1 in both wildtype and *Setdb1* cKO) are shown separately in **B**. Grey: autosomal genes. Orange: X chromosome genes. Blue: Y chromosome genes. TSS: transcription start site. TES: transcription end site.

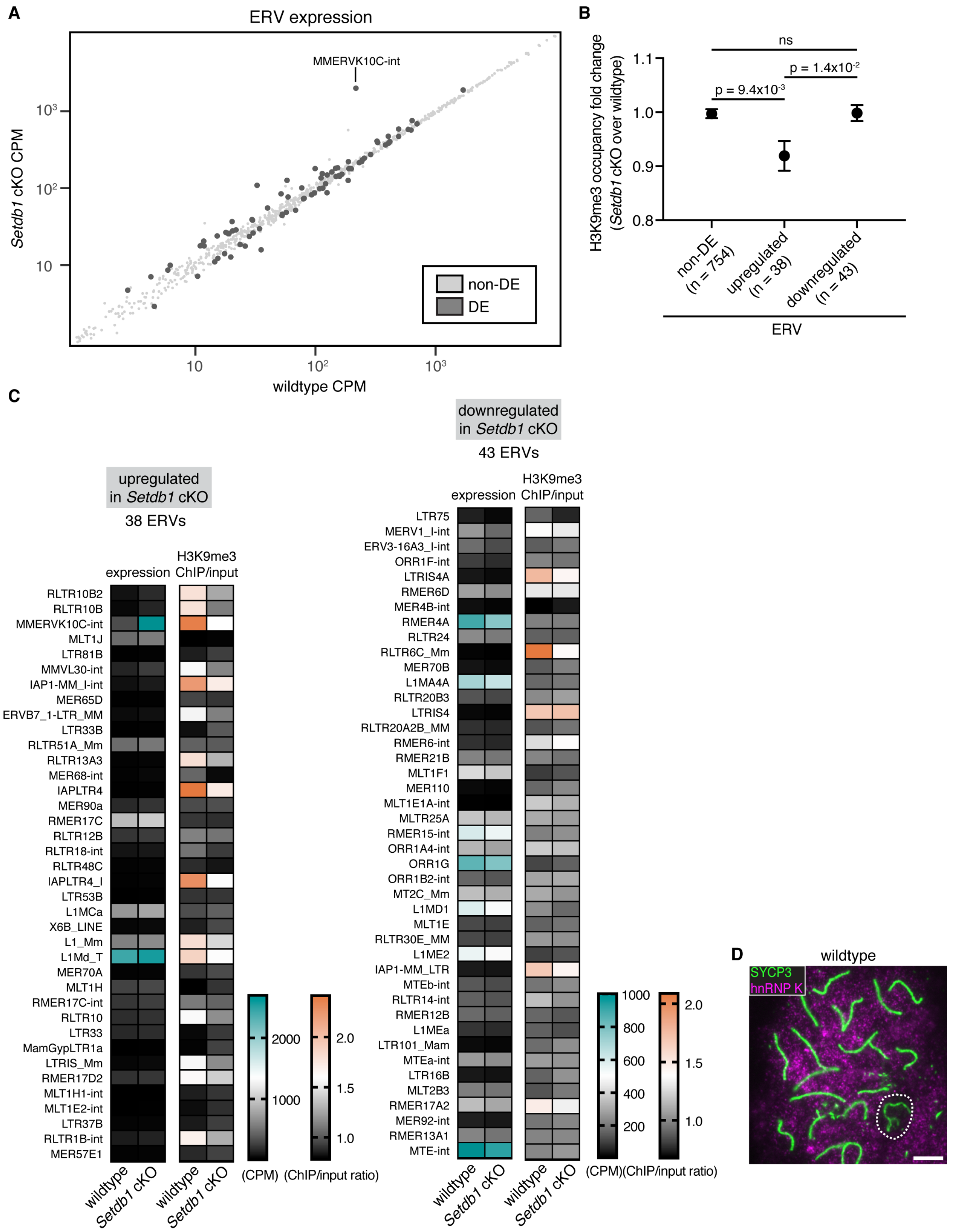

Figure S7. ERV expression in the *Setdb1* cKO and hnRNP K localisation in wildtype pachytene cells (related to Figures 5 and 6)

(**A**) Comparison of ERV expression levels between wildtype and *Setdb1* cKO pachytene cells. DE: differentially expressed. Light grey: non-DE ERVs. Dark grey: DE ERVs. CPM: counts per million.

(**B**) H3K9me3 occupancy fold-change of *Setdb1* cKO relative to wildtype in non-DE, upregulated, and downregulated ERVs. Chart shows mean fold change ± standard error. p-value calculated using Welch's t-test. ns: not significant.

(**C**) Heatmaps show CPM of ERV expression and H3K9me3-ChIP/input CPM ratio in upregulated and downregulated genes in *Setdb1* cKO**.**

(**D**) Early pachytene spermatocytes from wildtype (n = 30 cells) immunostained for SYCP3 (green) and hnRNP K (magenta). Dashed circle: XY pair. 8-week old mice were used for analysis. Scale bar: 5 μm.

See also Table S3.
